# Microglia and CD8^+^ T cell activation precede neuronal loss in a murine model of spastic paraplegia 15

**DOI:** 10.1084/jem.20232357

**Published:** 2025-04-23

**Authors:** Aleksej Frolov, Hao Huang, Dagmar Schütz, Maren Köhne, Nelli Blank-Stein, Collins Osei-Sarpong, Maren Büttner, Tarek Elmzzahi, Mukhran Khundadze, Marina Zahid, Michael Reuter, Matthias Becker, Elena De Domenico, Lorenzo Bonaguro, Axel Kallies, Helen Morrison, Christian A. Hübner, Kristian Händler, Ralf Stumm, Elvira Mass, Marc D. Beyer

**Affiliations:** 1 Immunogenomics and Neurodegeneration, Deutsches Zentrum für Neurodegenerative Erkrankungen (DZNE), Bonn, Germany; 2 Systems Medicine, Deutsches Zentrum für Neurodegenerative Erkrankungen (DZNE), Bonn, Germany; 3 Department of Microbiology and Immunology, The Peter Doherty Institute for Infection and Immunity, University of Melbourne, Melbourne, Australia; 4 Developmental Biology of the Immune System, Life & Medical Sciences (LIMES) Institute, University of Bonn, Bonn, Germany; 5 Institute of Pharmacology and Toxicology, Jena University Hospital, Friedrich-Schiller-University Jena, Jena, Germany; 6 Institute of Experimental Pathology, Centre of Molecular Biology of Inflammation, University of Münster, Münster, Germany; 7 Genomics and Immunoregulation, Life & Medical Sciences (LIMES) Institute, University of Bonn, Bonn, Germany; 8 Institute of Human Genetics, Jena University Hospital, Friedrich-Schiller-University Jena, Jena, Germany; 9 Center for Rare Diseases, University Hospital Jena, Friedrich-Schiller-University, Jena, Germany; 10 Institute of Laboratory Medicine, Clinical Chemistry and Pathobiochemistry, Charité - Universitätsmedizin Berlin, Berlin, Germany; 11 Leibniz Institute on Aging, Fritz Lipmann Institute, Jena, Germany; 12 Modular High-Performance Computing and Artificial Intelligence, Deutsches Zentrum für Neurodegenerative Erkrankungen (DZNE), Bonn, Germany; 13 PRECISE Platform for Single Cell Genomics and Epigenomics, DZNE and University of Bonn and West German Genome Center, Bonn, Germany; 14 Faculty of Biological Sciences, Friedrich-Schiller University, Jena, Germany; 15 Institute of Human Genetics, Universitätsklinikum Schleswig-Holstein, University of Lübeck and University of Kiel, Lübeck, Germany

## Abstract

In central nervous system (CNS) diseases characterized by late-onset neurodegeneration, the interplay between innate and adaptive immune responses remains poorly understood. This knowledge gap is exacerbated by the prolonged protracted disease course as it complicates the delineation of brain-resident and infiltrating cells. Here, we conducted comprehensive profiling of innate and adaptive immune cells in a murine model of spastic paraplegia 15 (SPG15), a complicated form of hereditary spastic paraplegia. Using fate-mapping of bone marrow–derived cells, we identified microgliosis accompanied by infiltration and local expansion of T cells in the CNS of *Spg15*^*−/−*^ mice. Single-cell analysis revealed an expansion of disease-associated microglia (DAM) and effector CD8^+^ T cells prior to neuronal loss. Analysis of potential cell–cell communication pathways suggested bidirectional interactions between DAM and effector CD8^+^ T cells, potentially contributing to disease progression in *Spg15*^*−/−*^ mice. In summary, we identified a shift in microglial phenotypes associated with the recruitment and expansion of T cells as a new characteristic of *Spg15*-driven neuropathology.

## Teaser

Frolov et al. discover an expansion and activation of microglia and T cells preceding neuronal loss in spastic paraplegia 15, highlighting the importance of immune responses and myeloid cell–lymphocyte interactions in the pathogenesis of hereditary spastic paraplegias.

## Graphical Abstract

**Figure:**
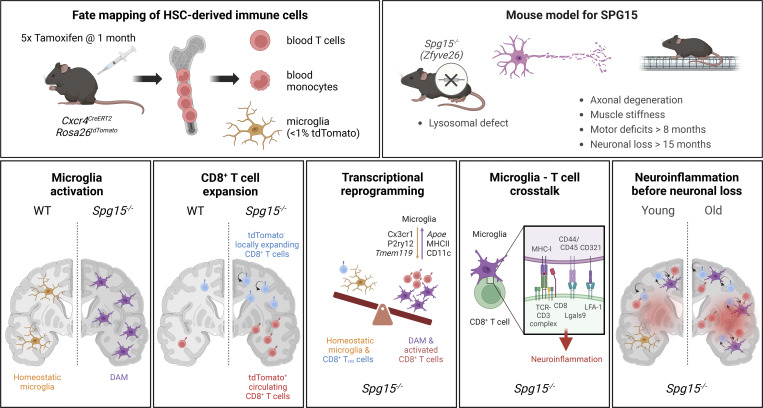

## Introduction

Hereditary spastic paraplegias (HSPs) are a group of genetic disorders marked by corticospinal tract dysfunction, leading to progressive gait disturbance and lower limb spasticity (Fereshtehnejad et al., 2023; McDermott et al., 2000). Over 80 spastic paraplegia genes (SPGs) have been identified, affecting membrane trafficking, mitochondrial function, metabolism, organelle biogenesis, and myelination (Blackstone, 2018). A prominent neuropathological feature of HSPs is corticospinal axonal degeneration, with cortical layer V motor neuron loss likely occuring secondary to axonal damage (McDermott et al., 2000). Recent studies in murine models highlight the role of neuroinflammation in HSP. Mice with proteolipid protein-1 gene (*Plp1*) mutations (*Spg2*) exhibit disease-amplifying neuroinflammation (Groh et al., 2016), while *Spg11*-deficient mice show microgliosis, CD8^+^ T cell accumulation, and motor impairment, mimicking SPG11 patients (Hörner et al., 2022). This growing evidence implicating neuroinflammation in HSP pathogenesis (Groh et al., 2016; Branchu et al., 2017; Hörner et al., 2022; Krumm et al., 2024) necessitates single-cell characterization of microglia and T cells. This approach can identify transcriptional differentiation and functional specialization, as seen in aging and neurodegeneration (Kaya et al., 2022; Chen and Colonna, 2021; Chen et al., 2023). Additionally, it enables the detection of disease-associated microglia (DAM), a key state in neuroinflammation (Deczkowska et al., 2018; Paolicelli et al., 2022; Keren-;Shaul et al., 2017). Microglial activation can trigger adaptive immune cell influx, exacerbating neuroinflammation. While CD8^+^ T effector memory (Tem) cells may support homeostatic microglial functions (Ritzel et al., 2016), expanded IFNγ^+^ PD1^+^ CD8^+^ T cells impair neural stem cells (Dulken et al., 2019) and drive microglia into a DAM-like state, worsening neurodegeneration (Kaya et al., 2022; Chen et al., 2023).

Here, we examine neuroinflammation in a murine model of HSP type 15 (*Spg15*^*−/−*^) (Khundadze et al., 2013), mimicking SPG15 loss-of-function mutations found in humans (Saffari et al., 2023). *SPG15* (*ZFYVE26*) encodes Spastizin, which interacts with Spatacsin (SPG11) and cooperates with the fifth adaptor protein complex (AP5) in endosomal trafficking, lysosomal biogenesis, and autophagy (Pozner et al., 2020). While both proteins operate in similar processes, they have distinct roles in autophagy and endocytosis (Vantaggiato et al., 2019; Khundadze et al., 2021). *Spg15*^−/−^ mice develop progressive motor deficits and neurodegeneration, mirroring human pathology (Khundadze et al., 2013). Clinically, SPG15 and SPG11 are indistinguishable, presenting with childhood cognitive impairment and progressive spasticity (Pensato et al., 2014). Notably, SPG15 patients often experience speech and learning delays preceding motor symptoms (Saffari et al., 2023). However, the mechanisms underlying cognitive and motor impairments in complicated HSPs remain poorly understood.

Despite their relevance, interactions between innate and adaptive immune cells in late-onset neurodegeneration remain underexplored. To address this, we employed *Cxcr4*^*CreERT2*^-mediated fate mapping in *Spg15*^−/−^ mice to track hematopoietic stem cell progeny in the inflamed brain (Werner et al., 2020). Combining this model with immunohistology and multiomic analysis, we provide the first comprehensive assessment of CNS immune responses in *Spg15*^−/−^ mice, advancing our understanding of complicated HSP.

## Results and discussion

### Microglia activation precedes neuronal cell loss in *Spg15*^*−/−*^ mice

**Figure 1.**
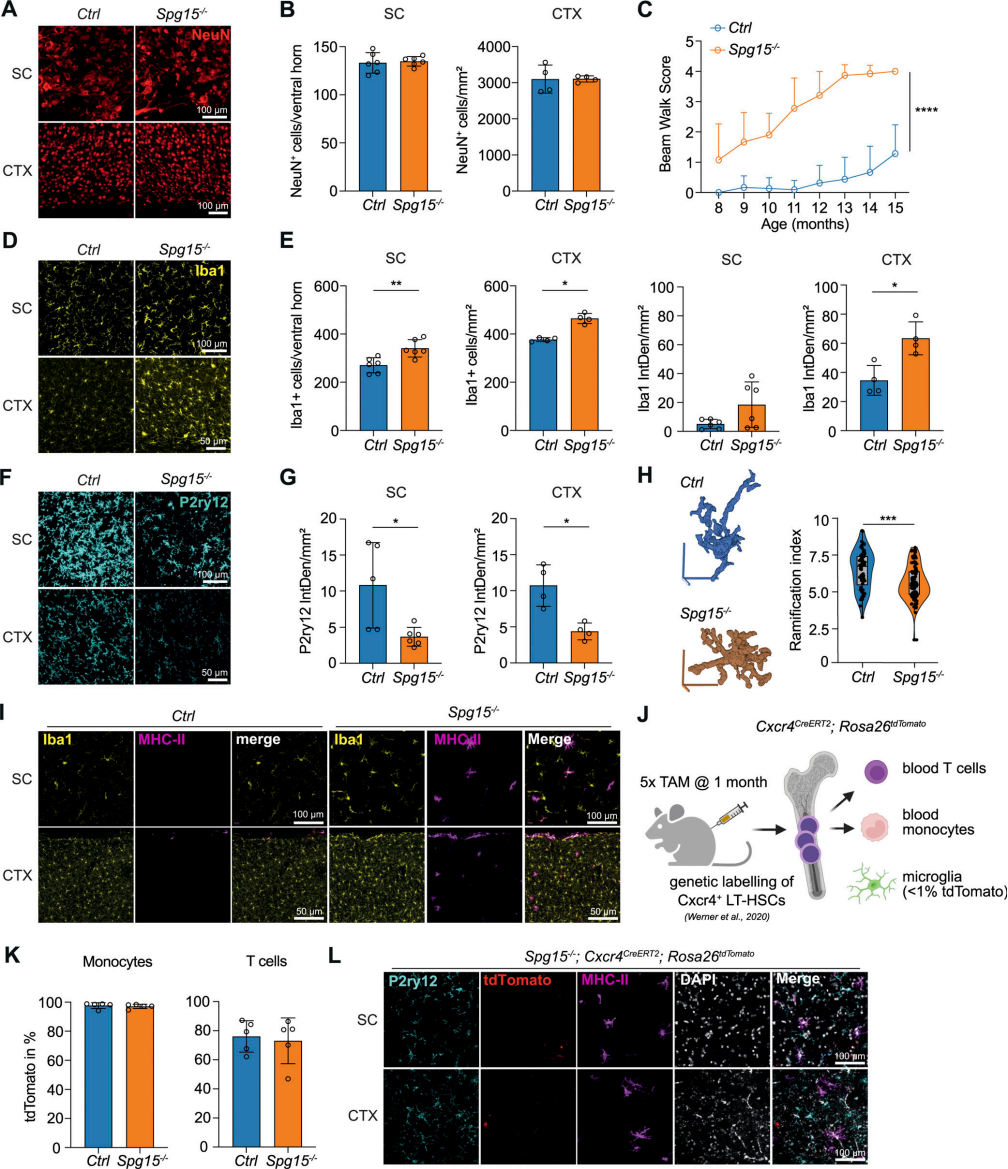
**Microglial activation in the CNS of *Spg15*** ^***−/−***^ **mice. (A and B)** Representative immunofluorescent (IF) staining for NeuN (A) and quantification of NeuN^+^ neurons (B) in the ventral horn of the SC, as cells per ventral horn; and layers V/VI of the primary motor cortex (CTX), as cells per mm^2^, in old (12–15 mo) control (*Ctrl*) and *Spg15*^*−/−*^ mice. *n* = 6 mice per genotype on two experimental days. **(C)** Motor impairment of *Ctrl* and *Spg15*^*−/−*^ mice at the indicated age as assessed by beam walk test. *n* = 7–23 per time point and genotype. **(D and E)** Representative IF staining for Iba1 (D), as well as quantification of Iba1^+^ cells per ventral horn (SC) or mm^2^ (CTX) and of Iba1 IF signal as integrated density (IntDen) per mm^2^ (E) in SC and CTX of old *Ctrl* and *Spg15*^*−/−*^ mice. *n* = 4–6 mice per genotype on two experimental days. **(F and G)** Representative IF staining for P2ry12 (F) and quantification of P2ry12 IF signal as IntDen per mm^2^ (G) in SC and CTX of old *Ctrl* and *Spg15*^*−/−*^ mice. **(H)** MotiQ morphometric analysis of the ramification of Iba1^+^ cells in the dorsal thalamus of old *Ctrl* and *Spg15*^*−/−*^ mice. The 3D pictures show representative Iba1^+^ cells analyzed for their ramification index after reconstruction from confocal z stacks. 60 cells are analyzed from *n* = 3 *Ctrl* mice and 95 cells from *n* = 4 *Spg15*^*−/−*^ mice on 2 experimental days. **(I)** Representative IF staining for MHC-II and Iba1 in SC and CTX from old *Ctrl* and *Spg15*^*−/−*^ mice. **(J–L)** Scheme of the *Cxcr4*^*CreERT2*^; *Rosa26*^*tdTomato*^ fate-mapping model used in K and L (created with https://BioRender.com). Mice were injected with tamoxifen (TAM) at 4 wk of age, resulting in efficient labeling of long-term hematopoietic stem cells (LT-HSCs) and their progeny, but not microglia. **(K)** Labeling efficiency of Ly6C^+^ blood monocytes and circulating CD3^+^ T cells in old *Spg15*^*+/+*^; *Cxcr4*^*CreERT2*^; *Rosa26*^*tdTomato*^*(Ctrl)* and *Spg15*^*−/−*^; *Cxcr4*^*CreERT2*^; *Rosa26*^*tdTomato*^ (*Spg15*^*−/−*^) mice. **(L)** Representative IF staining for MHC-II, P2ry12, and tdTomato in SC and CTX from old *Spg15*^*−/−*^; *Cxcr4*^*CreERT2*^; *Rosa26*^*tdTomato*^ mice. **(J–L)***n* = 5 mice per genotype on two experimental days. Data are shown as mean with SD in B, C, E, G, and K. Statistical significance was assessed with a Mann–Whitney *U* test in B, E, G, and K, Wilcoxon rank sum exact test in H, and mixed-effects analysis in C, and *P < 0.05, **P < 0.01, ***P < 0.001, ****P < 0.0001.

**Figure S1.**
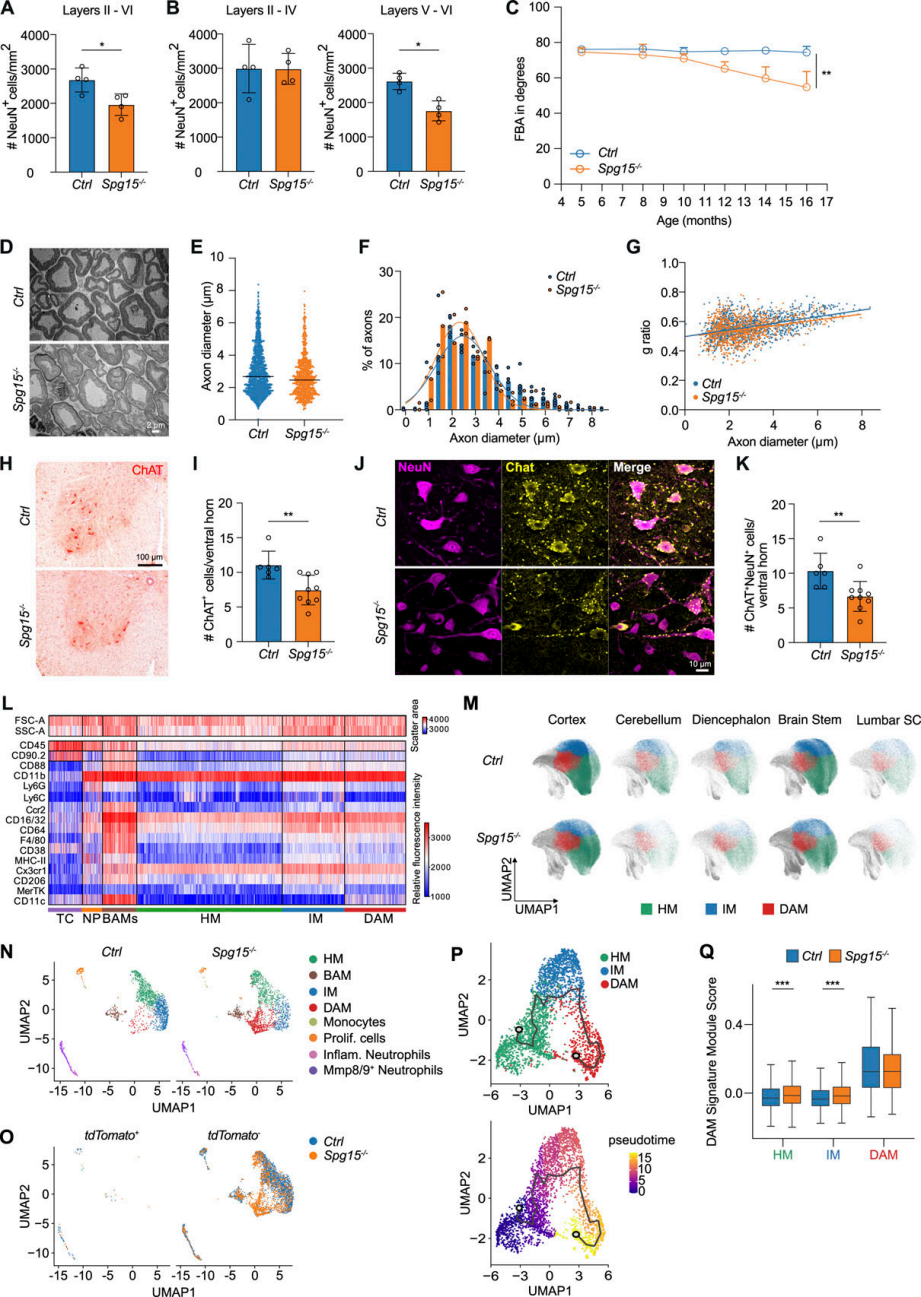
**Neuronal loss and immunophenotyping of microglia in the CNS of old *Spg15*** ^***−/−***^ **mice. (A and B)** Quantification of NeuN^+^ neurons shown as number per mm^2^ in layers II–VI (A), II–IV (B, left), and V–VI (B, right) of the cortex from 15- to 18-mo-old *Ctrl* and *Spg15*^*−/−*^ mice. *n* = 4 mice per genotype on two experimental days. **(C)** Motor impairment of *Ctrl* and *Spg15*^*−/−*^ mice at the indicated ages assessed by foot-base angle (FBA) test. *n* = 10 mice per time-point and genotype. **(D)** Representative electron microscopic pictures of sciatic nerves from 12- to 15-mo-old *Ctrl* and *Spg15*^*−/−*^ mice. **(E)** Average diameter of axons (μm) in *Ctrl* and *Spg15*^*−/−*^ mice. Each dot represents one axon. **(F)** Frequency distribution of axon diameters of sciatic nerves from 12- to 15-mo-old *Ctrl* and *Spg15*^*−/−*^ mice. Each mouse is represented as a dot. **(G)** Scatter plot depicting g ratio (y-axis) and respective axon diameter (x-axis) comparing 12–15-mo-old *Ctrl* (*n* = 5) and *Spg15*^*−/−*^ (*n* = 3) mice. **(D–G)***n* = 5 mice for *Ctrl* and *n* = 3 mice for *Spg15*^*−/−*^ on two experimental days. **(H)** Representative choline acetyltransferase (ChAT) staining of the ventral horn of 12–to 15-mo-old *Ctrl* and *Spg15*^*−/−*^ mice. **(I)** Quantification of ChAT^+^ cells per ventral horn of 12–15-mo-old *Ctrl* and *Spg15*^*−/−*^ mice. **(J)** Representative IF staining of alpha motor neurons (ChAT^+^ NeuN^+^) in the ventral horn of 12–15-mo-old *Ctrl* and *Spg15*^*−/−*^ mice. **(K)** Quantification of ChAT^+^ NeuN^+^ alpha motor neurons in the ventral horn from 12- to 15-mo-old *Ctrl* and *Spg15*^*−/−*^ mice. **(H–K)***n* = 6 mice for *Ctrl* and *n* = 9 mice for *Spg15*^*−/−*^ on two experimental days. Mean with SD is shown in A, B, I, and K, and the median is shown in E. Statistical significance was assessed by the Mann–Whitney *U* test in A, B, E, I, and K, and mixed-effects analysis in C, and *P < 0.05, **P < 0.01. **(L)** Fluorescence intensity in a linear (SSC/FSC) or biexponential scale (rest) of surface marker expression of single cells shown in Fig. 2 A. **(M)** UMAPs showing microglia clusters (homeostatic, HM; intermediate, IM; disease-associated, DAM) for single CNS regions from *Spg15*^*+/+*^; *Cxcr4*^*CreERT2*^; *Rosa26*^*tdTomato*^ (*Ctrl*) and *Spg15*^*−/−*^; *Cxcr4*^*CreERT2*^; *Rosa26*^*tdTomato*^ (*Spg15*^*−/−*^) mice. Combined clusters are depicted in Fig. 2 A. **(L and M)** 855,105 CD45^+^ cells from *n* = 3–6 mice per age and genotype on five experimental days. **(N)** UMAPs of scRNA-seq data split by old *Ctrl* and *Spg15*^*−/−*^ brains. Combined data are depicted in Fig. 3 A. **(O)** UMAP from Fig. 3 A split into tdTomato^+^ and tdTomato^−^ cells. **(N and O)** 5,378 CD11b^+^ cells from *n* = 3 mice per genotype on one experimental day. **(P)** Trajectory analysis of microglia using Monocle3. The trajectory path from HM to DAM is superimposed on the UMAP generated on microglia only (left). UMAP colored by pseudotime shows the distance to the defined starting node (labeled as 1) in the HM cluster (right). **(Q)** The module score for a DAM gene signature was calculated for each cell in the microglia subclusters. The distribution of module scores for each microglia subcluster was visualized using a boxplot. Outer boundaries represent the 75th or 25th percentile, respectively. The middle line represents the median. Whiskers indicate extreme values 1.5 times the interquartile range smaller or larger than the respective percentile. A double-sided Wilcoxon rank sum test in combination with the Holm method for multiple test correction was used to test differential enrichment of the DAM signature in *SPG15*^*−/−*^ versus *Ctrl* animals for each microglia subcluster, and ***P < 0.001. BAM: border-associated macrophages; DAM: disease-associated microglia; HM: homeostatic microglia; IM; intermediate microglia; Mmp8/9 NP: Mmp8/9^+^ neutrophils; Inflam. NP: proinflammatory neutrophils; MC: monocytes; PC: proliferating cells.

A previous study in 15-mo-old *Spg15*^*−/−*^ mice reported astrogliosis across brain regions (Marrone et al., 2022). Since microglial activation typically precedes astrocyte reactivity (Liddelow et al., 2017), we hypothesized that *Spg15*-deficiency triggers immune cell activation, contributing to neurodegeneration. To address a possible immune cell involvement in neuronal loss observed in 15-mo-old *Spg15*^*−/−*^ mice (Marrone et al., 2022), we quantified neurons in the spinal cord (SC) and cortex of younger animals. At 12–15 mo, *Spg15*^*−/−*^ mice showed no significant NeuN^+^ cell loss in the ventral horn of the lumbar SC or layers V/VI of the primary motor cortex (Fig. 1, A and B). However, neuronal loss was evident in 15- to 18-mo-old mice in cortical layers V/VI (Fig. S1, A and B) (Khundadze et al., 2013; Marrone et al., 2022). Intriguingly, *Spg15*^*−/−*^ mice developed gait abnormalities as early as 8–10 mo, assessed via beam walk and foot-base angle tests (Fig. 1 C and Fig. S1 C). To investigate the progressive neuronal dysfunction, we examined neuronal axon diameters in the sciatic nerve using electron microscopy at 12–15 mo, finding no genotype differences in axon size or myelination (Fig. S1, D–G). However, we detected a reduction in cholinergic motor neurons (choline acetyltransferase, ChAT^+^ motor neurons) in the ventral horn of the lumbar SC (Fig. S1, H and I), specifically affecting alpha motor neurons (ChAT^+^ NeuN^+^) (Fig. S1, J and K). Given that up to 40% of murine spinal motor neurons may be lost without causing signs of muscle weakness (Simon et al., 2010), motor deficits in *Spg15*^*−/−*^ mice are most likely not only a result of late-onset motor neuron loss.

Since many neurodegenerative diseases are accompanied by neuroinflammation and microglial activation (Paolicelli et al., 2022), we assessed microglial activation by immunofluorescence. Iba1^+^ signal and cell numbers were increased in the SC and cortex (Fig. 1, D and E). In the SC, white matter was most affected, while in the brain, multiple regions were affected, including the motor cortex, dorsal thalamus, and hippocampus (data not shown). These observations were supported by quantification of Iba1 and P2ry12 signals (Fig. 1, D–G), indicative of widespread microglial activation (Mildner et al., 2017; Haynes et al., 2006; Ito et al., 1998). MotiQ analysis (Hansen et al., 2022) revealed that *Spg15*^*−/−*^ microglia were less ramified in the dorsal thalamus (Fig. 1 H), further supporting an activated state of microglia in the sensory brain region.

MHC-II expression, which is often upregulated by activated microglia and recruited monocyte-derived macrophages during neuroinflammation (Chen and Colonna, 2021), was detected in a subset of Iba1^+^ cells in the *Spg15*^*−/−*^ CNS parenchyma (shown for cortex and SC in Fig. 1 I) but restricted to meningeal border–associated macrophages (BAM) in controls (Mrdjen et al., 2018). To address whether MHC-II expression in Iba1^+^ cells originated from recruited monocyte-derived macrophages, we crossed the *Cxcr4*^*CreERT2*^*; Rosa26*^*tdTomato*^ fate-mapper with *Spg15*^*−/−*^ (hereafter called *Spg15*^*−/−*^; *Cxcr4*^*CreERT2*^; *Rosa26*^*tdTomato*^) (Fig. 1 J) to detect HSC-derived cells (Werner et al., 2020). Tamoxifen injection at 4 wk of age resulted in almost 100% labeling efficiency in monocytes in 12- to 15-mo-old animals (Fig. 1 K). Immunofluorescent staining of MHC-II, Iba1, and P2ry12 revealed MHC-II^+^Iba1^+^ and MHC-II^+^P2ry12^low^ cells were yolk sac–derived microglia and not of monocytic origin (shown for P2ry12 in Fig. 1 L). In summary, our findings demonstrate that *Spg15*^*−/−*^ mice exhibit widespread microglial activation before neuronal loss, implicating neuroinflammation in disease progression.

### Yolk sac**–**derived microglia acquire a DAM-like phenotype throughout the CNS of *Spg15*^*−/−*^ mice

**Figure 2.**
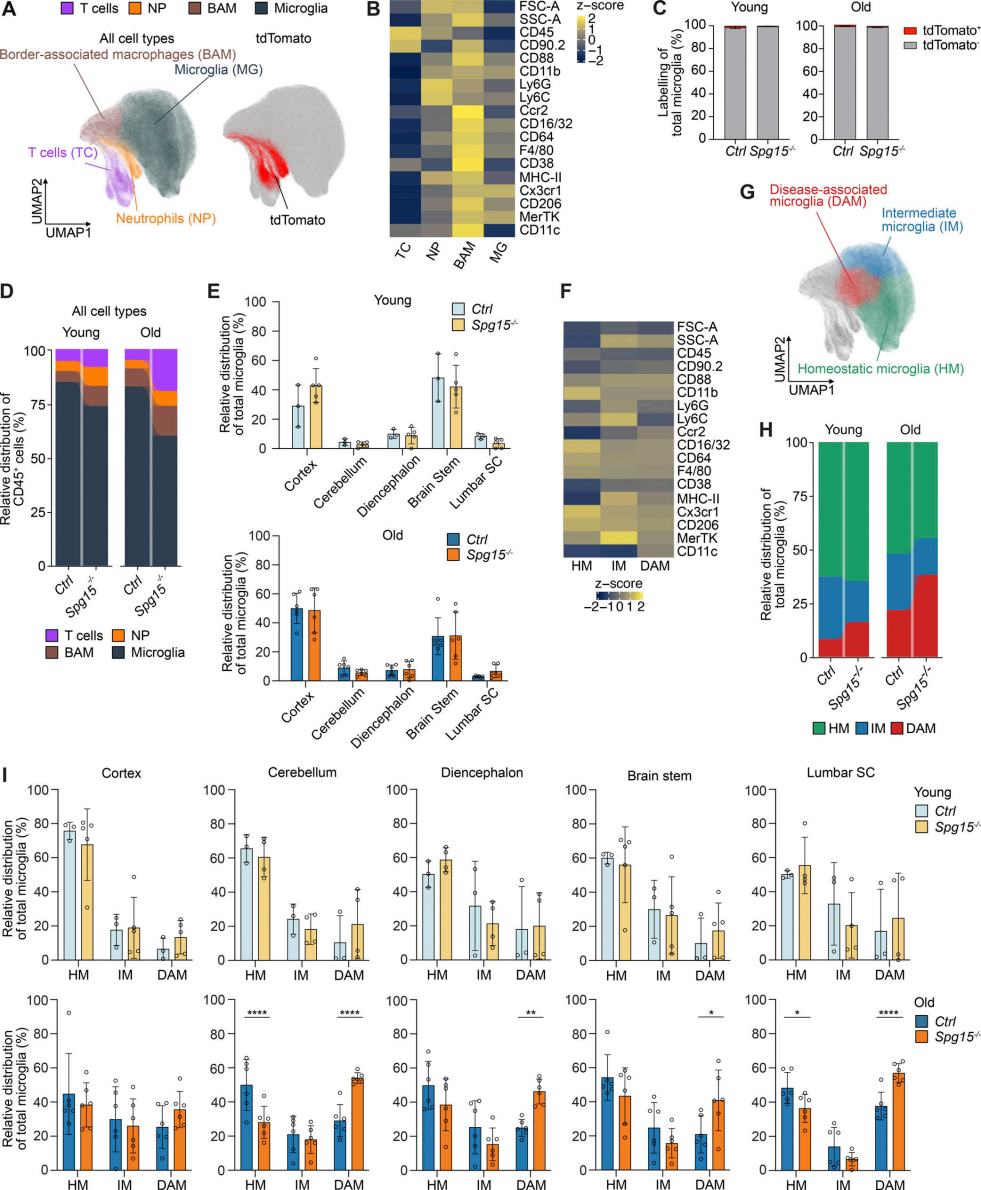
**Characterization of the immune cell landscape in the CNS of young and old Spg15** ^**−/−**^ **mice. (A)** Left: Unsupervised UMAP plot of a total of 855,105 CD45^+^ cells from young and old *Spg15*^*+/+*^; *Cxcr4*^*CreERT2*^; *Rosa26*^*tdTomato*^ (*Ctrl*) and *Spg15*^*−/−*^; *Cxcr4*^*CreERT2*^; *Rosa26*^*tdTomato*^ (*Spg15*^*−/−*^) mice, isolated from various regions across the CNS including brain stem, cerebellum, cerebral cortex, diencephalon, and lumbar SC. Cells were manually assigned into four cell types or cell states based on their surface marker expression. Right: tdTomato^+^ cells are displayed on the UMAP plot including all CD45^+^ cells. **(B)** Pseudobulk heatmap of surface markers detected in flow cytometry analysis determining cell types and subtypes. **(C)** tdTomato-labeling of microglia in young (left) and old (right) *Spg15*^*+/+*^; *Cxcr4*^*CreERT2*^; *Rosa26*^*tdTomato*^ (*Ctrl*) and *Spg15*^*−/−*^; *Cxcr4*^*CreERT2*^; *Rosa26*^*tdTomato*^ (*Spg15*^*−/−*^) mice. **(D)** Percentage of cell types or cell subtypes as defined in A of total CD45^+^ cells in either young or old mouse brains separated by genotypes. **(E)** Contribution of different CNS regions (brain stem, cerebellum, cortex, diencephalon, lumbar SC) to relative microglia numbers in either young (upper panel) or old (lower panel) *Ctrl* or *Spg15*^*−/−*^ mice. **(F)** Pseudobulk heatmap of surface markers detected in flow cytometry for microglia subtypes. **(G)** UMAP plot of microglia subtypes subsetted from A. **(H)** Percentage of microglia subtypes in all microglia in either young or old mouse CNS separated by genotypes. **(I)** Percentage of microglia subtypes in either young (upper panel) or old (lower panel) animals across CNS regions. *n* = 3–6 mice per age and genotype on five experimental days. Data are represented as mean with SD. Statistical significance was assessed with a Mann–Whitney *U* test in C and a two-way FDR-corrected ANOVA in E and I, and *P < 0.05, **P < 0.01, ****P < 0.0001. BAM: border-associated macrophages; DAM: disease-associated microglia; HM: homeostatic microglia; IM; intermediate microglia; NP: neutrophils.

Next, we analyzed immune cell populations in young (2–3 mo) and old (12–15 mo) *Spg15*^*−/−*^; *Cxcr4*^*CreERT2*^; *Rosa26*^*tdTomato*^ mice to characterize the contribution of resident and recruited immune cells to disease onset and progression. Using Uniform Manifold Approximation and Projection (UMAP) representation of flow cytometry data from 855,105 CD45^+^ cells across CNS regions (cerebral cortex, diencephalon, brain stem, cerebellum, and lumbar SC), we overlaid tdTomato signals (Fig. S1 L and Fig. 2 A). T cells (CD11b^−^CD90.2^+^), neutrophils (Ly6G^+^), BAMs (CD38^+^CD206^+^), and microglia (CD45^low^SSC-A^low^FSC-A^low^) were identified by marker expression and cell size (Fig. 2 B). As expected, most neutrophils expressed tdTomato (Fig. 2 A). In contrast, T cells consisted of tdTomato^+^ and tdTomato^−^ cells, while BAMs and microglia showed minimal tdTomato expression (Fig. 2, A and C). We categorized CD45^+^ cells by age and genotype, finding stable neutrophil and BAM proportions, while *Spg15*^*−/−*^ mice showed a decline in microglia abundance due to T cell expansion during disease (Fig. 2 D). Flow cytometry revealed no regional differences in the relative microglia distribution between control and *Spg15*^*−/−*^ mice (Fig. 2 E). These results, together with our immunohistological data showing microglia expansion in the cortex and SC (Fig. 1, D and E), support global microglia expansion in *Spg15*^*−/−*^ mice.

To explore microglial heterogeneity in *Spg15*^*−/−*^ mice, we used surface markers and cell size/granularity to further subcategorize microglia (Fig. S1 L and Fig. 2 F). We found three microglial subpopulations, which we termed homeostatic microglia (HM, Cx3cr1^high^), intermediate microglia (IM, increased size, granularity, and MHC-II expression), and DAM (MHC-II^+^ CD11c^+^) (Fig. 2, F and G; and Fig. S1 L). DAM were expanded in *Spg15*^*−/−*^ mice at 2–3 mo and increased further at 12–15 mo (Fig. 2 H).

Regional analysis showed DAM expansion in multiple CNS areas of old *Spg15*^*−/−*^ mice but not in young mice (Fig. 2 I and Fig. S1 M). These findings indicate a widespread conversion of HM to DAM in old *Spg15*^*−/−*^ mice before neuronal loss, mirroring changes observed in Alzheimer's disease models (Chen and Colonna, 2021; Deczkowska et al., 2018).

### *Spg15* ^*−/−*^ microglia transition into a proinflammatory state

**Figure 3.**
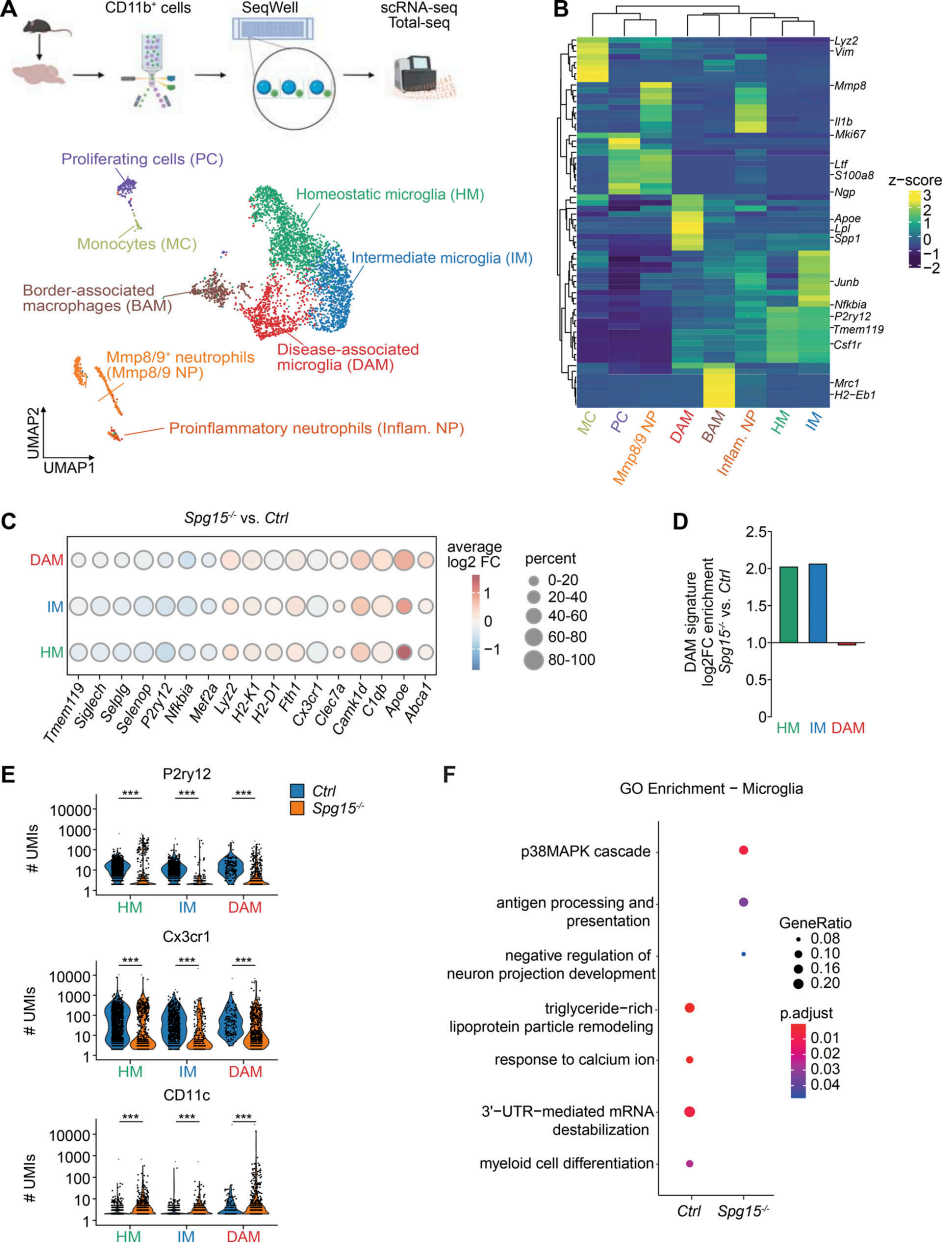
***Spg15*** ^***−/−***^ **microglia transition into DAM. (A)** Top: Scheme showing the isolation procedure and scRNA-seq of CD11b^+^ cells. Created with https://BioRender.com. Bottom: UMAP plot showing 5,378 CD11b^+^ cells isolated by flow cytometry from old *Spg15*^*+/+*^; *Cxcr4*^*CreERT2*^; *Rosa26*^*tdTomato*^ (*Ctrl*) and *Spg15*^*−/−*^; *Cxcr4*^*CreERT2*^; *Rosa26*^*tdTomato*^ (*Spg15*^*−/−*^) mice. Cells were manually assigned to eight cell types or cell states based on gene expression. **(B)** Pseudobulk heatmap of differentially expressed genes (DEGs) between clusters shown in A. DEGs were identified using the Wilcoxon rank-sum test. Resulting genes were filtered for a log_2_ fold change >0.25 and genes expressed in at least 10% of cells in the respective clusters. Canonical markers used for annotation of clusters are highlighted. **(C)** DEGs across microglia subpopulations comparing *Spg15*^*−/−*^ to *Ctrl* brains (P.adjust < 0.05). Log_2_ fold-change (log_2_FC) is indicated by red (upregulated in *Spg15*^*−/−*^) or blue (downregulated in *Spg15*^*−/−*^). The percentage of cells expressing the respective gene in each cell cluster is represented by the size of the expression circles. **(D)** Module score for a DAM gene signature obtained from Hammond et al. (2019) was calculated for each cell in the microglia subclusters. log_2_FC were calculated comparing *Spg15*^*−/−*^ to the median of the *Ctrl*. **(E)** Protein expression based on the number of UMIs aligned to the respective oligos in the Total-seq comparing microglia from *Ctrl* to *Spg15*^*−/−*^ mice. Statistical significance testing was performed using the Wilcoxon rank-sum test, and ***P < 0.001. **(F)** GOEA performed on DEGs from B. A hypergeometric test with all genes in the “biological process” database as background, for statistical enrichment testing, in combination with the Benjamini-Hochberg procedure for multiple testing correction was performed. Ontology terms were filtered for a q-value <0.2 and biological significance. *n* = 3 mice per genotype on one experimental day. BAM: border-associated macrophages; DAM: disease-associated microglia; HM: homeostatic microglia; IM; intermediate microglia; Mmp8/9 NP: Mmp8/9^+^ neutrophils; Inflam. NP: proinflammatory neutrophils; MC: monocytes; PC: proliferating cells.

To characterize the functional state of microglia in old *Spg15*^*−/−*^; *Cxcr4*^*CreERT2*^; *Rosa26*^*tdTomato*^ mice, we performed single-cell RNA-sequencing (scRNA-seq) of CD11b^+^ cells (Fig. 3 A) combined with a proteogenomics approach (Total-seq) for transcriptome and surface protein analysis. In the UMAP representation of the transcriptome data, we identified three microglia subpopulations with *Apoe*^high^*Lpl*^high^*Spp1*^*high*^ cells corresponding to DAM and *P2ry12*^*high*^*Tmem119*^*high*^*Csf1r*^*high*^ cells to HM and IM (Fig. 3, A and B). As expected, tdTomato transcripts were absent in microglia but detected in neutrophils and BAMs (Fig. S1, N and O). Pseudotime analysis (Cao et al., 2019) confirmed IM as a transitional state between HM and DAM (Fig. S1 P).

Differential expression analysis revealed that DAM from *Spg15*^*−/−*^ mice had higher levels of DAM-specific genes (*Apoe*, *C1qb*, and *Fth1*) compared with controls (Fig. 3 C) (Mathys et al., 2017; Deczkowska et al., 2018; Chen and Colonna, 2021). Notably, HM and IM from *Spg15*^*−/−*^ mice also upregulated DAM-associated genes, suggesting early microglial conversion (Fig. 3 C). A DAM signature score confirmed increased DAM-related gene expression in *Spg15*^*−/−*^ HM and IM (Fig. 3 D and Fig. S1 Q). Concurrently, homeostatic genes (*P2ry12*, *Tmem119*, and *Nfkbia*) were downregulated, mirroring the HM-to-DAM transition (Fig. 3 C) (Chen and Colonna, 2021). These data suggested that HM and IM were starting to convert into DAM-like cells. Total-seq analysis further validated these findings, showing decreased homeostatic markers (*P2ry12* and *Cx3cr1*) and increased CD11c (*Itgax*) in all *Spg15*^*−/−*^ microglia (Fig. 3 E).

Gene Ontology (GO) analysis revealed a downregulation of terms related to homeostasis (“response to calcium ion,” “triglyceride-rich lipoprotein particle remodeling”) and an upregulation of terms like “antigen processing and presentation,” in line with the observed MHC-II expression, as well as “p38MAPK cascade,” indicating that these cells undergo cellular stress (Fig. 3 F). Together, these multiomics findings confirm widespread microglial conversion toward a DAM-like phenotype in *Spg15*^*−/−*^ mice.

### Expansion of CD8^+^ T cells with an effector-like phenotype in the CNS of *Spg15*^*−/−*^ mice before onset of neuronal loss

**Figure 4.**
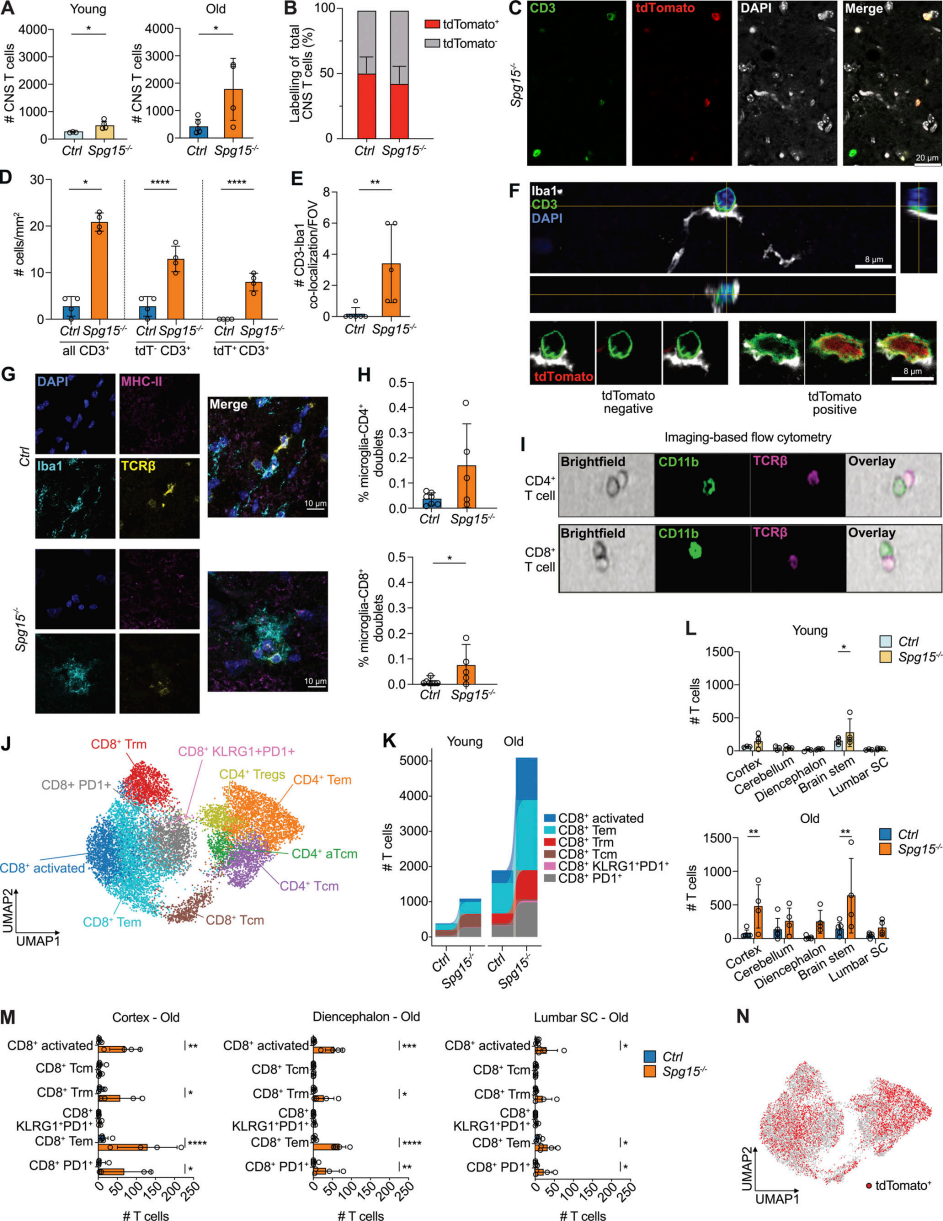
**High-dimensional analysis of the expanded T cell compartment in the CNS of *Spg15*** ^***−/−***^ **mice. (A)** Number of CNS CD3^+^ T cells in young (2–3 mo) and old (12–15 mo) *Spg15*^*+/+*^; *Cxcr4*^*CreERT2*^; *Rosa26*^*tdTomato*^ (*Ctrl*) and *Spg15*^*−/−*^; *Cxcr4*^*CreERT2*^; *Rosa26*^*tdTomato*^ (*Spg15*^*−/−*^) mice analyzed by flow cytometry. **(B)** Flow cytometry analysis of tdTomato expression in CD3^+^ T cells present in the CNS of old *Ctrl* and *Spg15*^*−/−*^ mice. **(A and B)***n* = 3–4 mice per genotype on four experimental days. **(C)** Representative IF double-staining for CD3 and tdTomato in the brain of 12–15-mo-old *Spg15*^*−/−*^; *Cxcr4*^*CreERT2*^; *Rosa26*^*tdTomato*^ mice. **(D)** Quantification of CD3^+^ cells per mm^2^ in the CTX of 12–15-mo-old *Ctrl* and *Spg15*^*−/−*^ mice. **(C and D)***n* = 4 mice per genotype on two experimental days. **(E)** Quantification of CD3^+^ cells directly interacting with Iba1^+^ cells in the SC of *Ctrl* and *Spg15*^*−/−*^ mice as shown per field of view (FOV). **(F)** Single confocal plane of z-stack demonstrating direct interaction of an Iba1^+^ microglial cell with a CD3^+^ T cell in the SC. Orthogonal views of the z-stack are shown on the right (YZ) and below (XZ) the single plane. Enlarged details at the bottom demonstrate contact of Iba1^+^ processes with a tdTomato^−^ CD3^+^ T cell (left) and a tdTomato^+^ CD3^+^ T cell (right). Images are from the SC of 12–15-mo-old *Spg15*^*−/−*^; *Cxcr4*^*CreERT2*^; *Rosa26*^*tdTomato*^ (*Spg15*^*−/−*^) mice. **(E and F)***n* = 5 mice per genotype on two experimental days. **(G)** Representative IF staining for Iba1, TCRβ and MHC-II in SC from 12- to 15-mo-old *Ctrl* and *Spg15*^*−/−*^ mice. **(H)** Percentage of microglia interacting with CD4^+^ (top) or CD8^+^ (bottom) T cells in the SC from 12- to 15-mo-old *Ctrl* and *Spg15*^*−/−*^ mice by imaging-based flow cytometry. **(I)** Representative pictures of interactions between CD45^low^ CD11b^+^ Cx3cr1^+^ microglia and TCRβ^+^ CD4^+^ (upper) or TCRβ^+^ CD8^+^ (lower) T cells in the SC from 12- to 15-mo-old *Ctrl* and *Spg15*^*−/−*^ mice by imaging-based flow cytometry. **(G–I)***n* = 7 mice for *Ctrl* and *n* = 5 *Spg15*^*−/−*^ on two experimental days. **(J)** Unsupervised UMAP of CD3^+^ T cell clusters from control and *Spg15*^*−/−*^ mouse brains. T cells were pooled from young and old mice and annotated according to key marker protein expression analyzed by flow cytometry. **(K)** Compositional changes of CD8^+^ T cell subtypes as defined in J in young and old *Ctrl* and *Spg15*^−/−^ mice. **(L)** Number of CD3^+^ T cells in different brain regions shown for young (top) and old (bottom) *Ctrl* and *Spg15*^−/−^ mice. **(M)** Compositional changes of CD3^+^ T cell subsets as defined in J in cortex (left), diencephalon (middle), and lumbar SC (right) of old control and *Spg15*^*−/−*^ mice. **(N)** tdTomato expression overlaid on the UMAP defined in J. **(J–N)***n* = 4–5 mice per age and genotype on five experimental days. Data are represented as mean with SD. Statistical significance was assessed with a Mann–Whitney *U* test in A, D (all CD3^+^), E, H, and L and a two-way FDR-corrected ANOVA in D (tdT^−^ CD3^+^ and tdT^+^ CD3^+^), M, and *P < 0.05, **P < 0.01, ***P < 0.001, ****P < 0.0001. Trm: resident memory T cells; Tem: effector memory T cells; Tcm: central memory T cells; CD4^+^ Tregs: CD4^+^ regulatory T cells; aTcm: activated central memory T cells.

**Figure S2.**
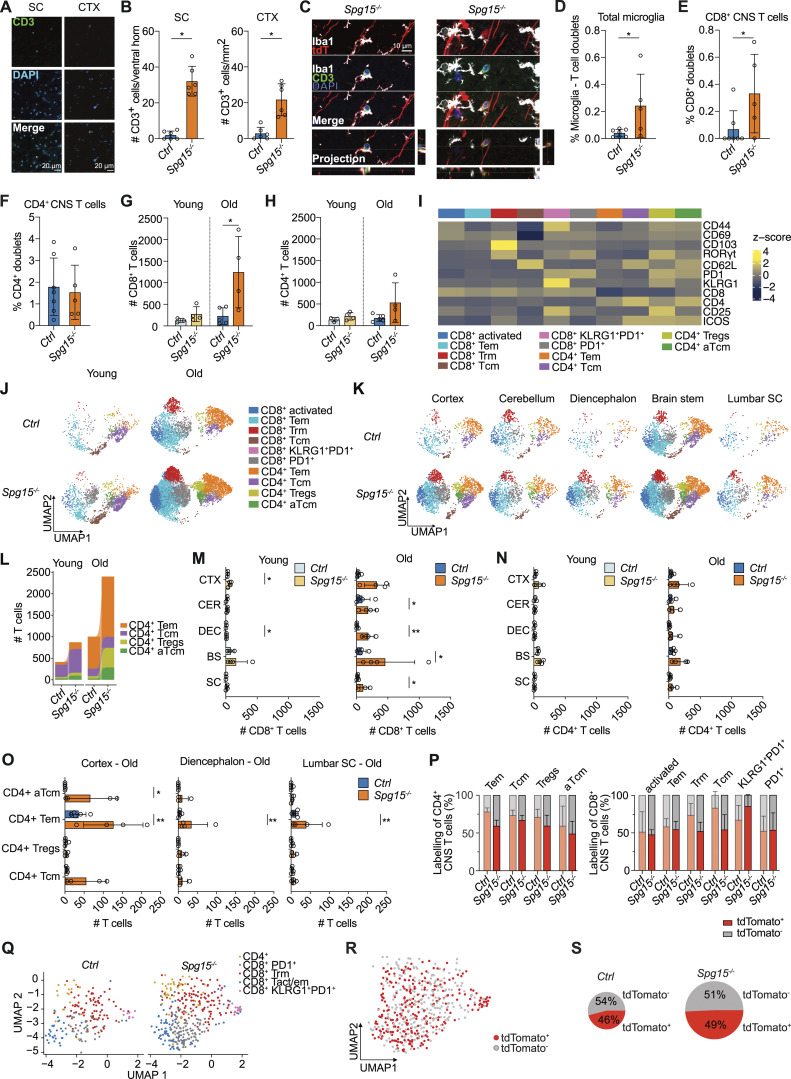
**Analysis of the enriched T cell compartment in the CNS of *Spg15*** ^***−/−***^ **mice. (A)** Representative IF staining for CD3 in spinal cord (SC) (left) and cortex (CTX) (right) from 12 to 15 mo old *Spg15*^*−/−*^; *Cxcr4*^*CreERT2*^*; Rosa26*^*tdTomato*^ (*Spg15*^*−/−*^) mice. **(B)** Quantification of CD3^+^ cells per mm^2^ in SC (left graph) and CTX (right graph) of *Ctrl* and *Spg15*^*−/−*^ mice aged 12–15 mo. **(A and B)***n* = 6 mice per genotype on two experimental days. **(C)** Confocal planes of z-stacks demonstrating direct interaction of Iba1^+^ microglial cells with a tdTomato^−^ (left) or tdTomato^+^ (right) CD3^+^ T cell in the SC. Orthogonal views of the z-stack are shown on the right (YZ) and below (XZ) the single plane. Images are from the SC of 12–15-mo-old *Spg15*^*−/−*^; *Cxcr4*^*CreERT2*^; *Rosa26*^*tdTomato*^ (*Spg15*^*−/−*^) mice. **(D–F)** Percentage of total CD3^+^ T (D), CD8^+^ T (E), or CD4^+^ T (F) cells interacting with microglia in SC from 12- to 15-mo-old *Ctrl* and *Spg15*^*−/−*^ mice quantified from imaging-based flow cytometry experiment C–F, *n* = 7 mice for *Ctrl* and *n* = 5 mice for *Spg15*^*−/−*^ on two experimental days. **(G and H)** Number of CD8^+^ (G) and CD4^+^ (H) T cells in the CNS of young (2–3 mo, left) and old (12–15 mo, right) *Ctrl* and *Spg15*^*−/−*^ mice analyzed by flow cytometry. **(I)** Pseudobulk heatmap of surface markers detected in flow cytometry analysis determining cell types and subtypes In Fig. 4 J. **(J)** Distribution of T cells after splitting the UMAP from Fig. 4 J according to genotype and age. **(K)** UMAPs showing T cells after splitting the UMAP from Fig. 4 J according to genotype and brain region. **(L)** Compositional changes of CD4^+^ T cell subtypes as defined in Fig. 4 J. **(M and N)** Number of CD8^+^ (M) and CD4^+^ (N) T cells in various brain regions of young (left) and old (right) *Spg15*^*+/+*^; *Cxcr4*^*CreERT2*^; *Rosa26*^*tdTomato*^ (*Ctrl*) and *Spg15*^*−/−*^; *Cxcr4*^*CreERT2*^; *Rosa26*^*tdTomato*^ (*Spg15*^*−/−*^) mice. (**G–N)***n* = 3–5 mice per age and genotype on five experimental days. CTX: Cortex; CER: Cerebellum; DEC: Diencephalon; BS: Brain stem; SC: Spinal cord. **(O)** Number of CD4^+^ T cells per T cell subset in 12–15-mo-old cortex (left), diencephalon (middle), and lumbar SC (right) of *Ctrl* (*n* = 5) and *Spg15*^*−/−*^ (*n* = 4). For definition of T cell subsets see Fig. 4 J. **(P)** tdTomato expression in CD3^+^ T cell subclusters present in the CNS of old (12–15 mo) *Ctrl* and *Spg15*^*−/−*^ mice. Left: subclusters found in CD4^+^ T cells. Right: subclusters found in CD8^+^ T cells. For the definition of cell types refer to legend in Fig. 4 J. **(O and P)***n* = 3–5 mice per age and genotype on five experimental days. **(Q)** UMAP from full length scRNA-seq data from CNS T cells isolated from old (12–15 mo) *Spg15*^*+/+*^; *Cxcr4*^*CreERT2*^; *Rosa26*^*tdTomato*^ (*Ctrl*) and *Spg15*^*−/−*^; *Cxcr4*^*CreERT2*^; *Rosa26*^*tdTomato*^ (*Spg15*^*−/−*^) animals split by genotype. **(R)** tdTomato expression in single T cells was mapped onto the UMAP from Fig. 5 A. tdTomato expression was extracted from index sorting data generated during sorting into 384-well plates. **(S)** Quantification of tdTomato^+^ or tdTomato^−^ in the full-length scRNA-seq data generated with Smart-Seq2 from CNS T cells isolated from the CNS of 12–15-mo-old *Ctrl* and *Spg15*^*−/−*^ mice. The percentage of either tdTomato^+^ (red) or tdTomato^−^ CD8^+^ T cells (grey) in old *Ctrl* or *Spg15*^*−/−*^ animals are shown as pie charts. The size of the pies represents the relative number of cells in the respective condition. **(Q–S)***n* = 4 mice for *Ctrl* and *n* = 3 for *Spg15*^*−/−*^ one experimental day. Data are represented as mean with SD in B, D–H, and M–O. Data are represented as mean with SEM in P. Statistical significance was assessed with a Mann–Whitney *U* test in B, D–H, M, and N. Statistical significance was assessed with a two-way FDR-corrected ANOVA in O, and *P < 0.05, **P < 0.01. Trm: resident memory T cells; Tem: effector memory T cells; Tcm: central memory T cells; CD4^+^ Tregs: CD4^+^ regulatory T cells; aTcm: activated central memory T cells; CD8^+^ activated: activated CD8^+^ T cells; CD8^+^ PD1^+^: PD1^+^ CD8^+^ T cells; CD8^+^ KLRG1^+^PD1^+^: KLRG1^+^PD1^+^ CD8^+^ T cells.

MHC-dependent antigen presentation is key for T cell activation in CNS inflammation (Berriat et al., 2023; Goverman, 2009). Given increased MHC-II^+^ microglia and T cells in *Spg15*^*−/−*^ mice (Fig. 1 and Fig. 2), we examined T cell infiltration in more detail. We observed increased numbers of CD3^+^ T cells in young and old *Spg15*^*−/−*^ mice by flow cytometry (Fig. 4 A) and confirmed this by immunostaining (Fig. S2, A and B). In the SC, CD3^+^ T cells were most abundant in white matter paralleling microglial activation (Fig. S2 A). Interestingly, while ∼80% of blood T cells were tdTomato^+^ (Fig. 1 K), only ∼50% of CNS-resident CD3^+^ T cells were labeled (Fig. 4 B) and we confirmed the presence and equal distribution of tdTomato^+^ and tdTomato^−^ CD3^+^ T cells via immunofluorescence (Fig. 4, C and D). These fate-mapping data indicate that microglia activation correlated with early recruitment and local expansion of tdTomato^−^ T cells and a later infiltration with tdTomato^+^ T cells generated from HSCs.

To investigate direct physical interactions between these two cell types, we used confocal microscopy and observed increased interactions of CD3^+^ T cells with microglial processes in Spg15^−/−^ mice (Fig. 4, E and F), which were detectable for both tdTomato^+^ and tdTomato^−^ T cells (Fig. 4 F and Fig. S2 C), indicating independence of the timepoint of T cell recruitment. In the SC, microglial filopodia were seen surrounding entire T cells in *Spg15*^−/−^ mice, a behavior absent in control mice (Fig. 4 G). Imaging flow cytometry revealed a higher frequency of T cell–microglia interactions in *Spg15*^−/−^ mice (Fig. S2 D), with both CD4^+^ and CD8^+^ T cells interacting more frequently with microglia (Fig. 4, H and I). Notably, there was a general increase in the frequency of CD8^+^ T cell-microglia doublets in *Spg15*^−/−^ brains, while CD4^+^ T cell–microglia doublets remained constant (Fig. S2, E and F). In summary, these data support that SPG15 pathology involves increased microglia–T cell interactions, driven predominantly by CD8^+^ T cells.

Flow cytometry revealed an expansion of both CD8^+^ and the CD4^+^ T cell in old *Spg15*^*−/−*^ mice with a pronounced increase in CD8^+^ T cell numbers (Fig. S2, G and H). To classify CNS T cells, we clustered all T cells based on canonical surface markers separating CD4^+^ and CD8^+^ T cell populations into phenotypically distinct subsets (Fig. 4 J and Fig. S2 I). Specifically, CD8^+^ T cells comprised six distinct subsets, namely CD62L^+^CD44^low^ central memory T cells (Tcm), and five populations of tissue-resident CD8^+^ T cells, namely CD44^+^CD69^+^CD62L^low^ effector memory T cells (Tem), CD44^high^CD25^+^CD69^+^ activated T cells (activated), CD103-expressing tissue-resident CD69^+^CD103^+^ T cells (Trm), PD1^+^CD69^+^CD103^−^ effector T cells (PD1^+^), and PD1^+^ T cells co-expressing KLRG1 (KLRG1^+^PD1^+^). CD4^+^ T cells included four subsets, CD4^+^CD25^high^ICOS^+^ Treg cells, CD62L^+^CD44^low^ central memory cells (Tcm), CD62L^low^CD44^+^ activated central memory cells (aTcm), and CD69^+/−^ effector memory cells (Tem) (Fig. 4 J). Comparing experimental groups (Fig. S2 J), we observed a shift from a central memory-like phenotype in young *Spg15*^−/−^ and control mice toward an effector-like state in old mice, more pronounced in *Spg15*^*−/−*^ mice (Fig. 4 K; and Fig. S2, K and L). This shift was driven by increased activated, Tem, Trm, and PD1^+^ effector CD8^+^ T cells (Fig. 4 K).

Based on distinct neuropathology and DAM-like microglia expansion (Fig. 2 I), we assessed T cell expansion across CNS regions. Old *Spg15*^−/−^ animals exhibited increased CD3^+^ T cells in all regions, with the highest enrichment in the cortex and diencephalon (Fig. 4 L; and Fig. S2, M and N). Subclassification revealed an expansion of activated, Tem, Trm, and PD1^+^ effector CD8^+^ T cells, consistent with findings for the entire CNS (Fig. 4 M). This suggests that CD8^+^ T cells contribute to pathology across the CNS, paralleling microglial activation. In contrast, the response within the CD4^+^ T cell compartment was more equivocal (Fig. S2, N and O).

Using the *Cxcr4*^*CreERT2*^ fate-mapping model, we quantified tdTomato^+^ T cells to distinguish local proliferation from CNS recruitment. tdTomato^+^ T cells were present in all clusters (Fig. 4 N), suggesting that T cell differentiation was independent of the time point of entry into the CNS. Analysis of all six CD8^+^ and four CD4^+^ T cell subpopulations in old *Spg15*^−/−^ mice revealed a distinct behavior. CD4^+^ T cells showed minimal differences, while CD8^+^KLRG1^+^PD1^+^ T cells in *Spg15*^−/−^ mice had a modest tdTomato^+^ increase (Fig. S2 P), indicating either higher recruitment or preferential local proliferation. Furthermore, substantial enrichment in tdTomato^−^ CD8^+^ Trm and Tcm cells (Fig. S2 P) suggests early CNS infiltration before tamoxifen treatment and subsequent local expansion.

Overall, CD8^+^ T cells with tissue-resident and effector phenotypes expand in *Spg15*^−/−^ mice. This expansion is already detectable at an early age but more prominent in old animals and involves both early-infiltrating tdTomato^−^ and late-infiltrating tdTomato^+^ CD8^+^ T cells. Like microglia, T cell activation extends beyond motor regions, potentially contributing to the complex neuropathology of SPG15 (Pensato et al., 2014).

### scRNA-seq of T cells in *Spg15*^*−/−*^ mice identifies a type-I interferon/PD1 co-expressing subpopulation of CD8^+^ T cells

**Figure 5.**
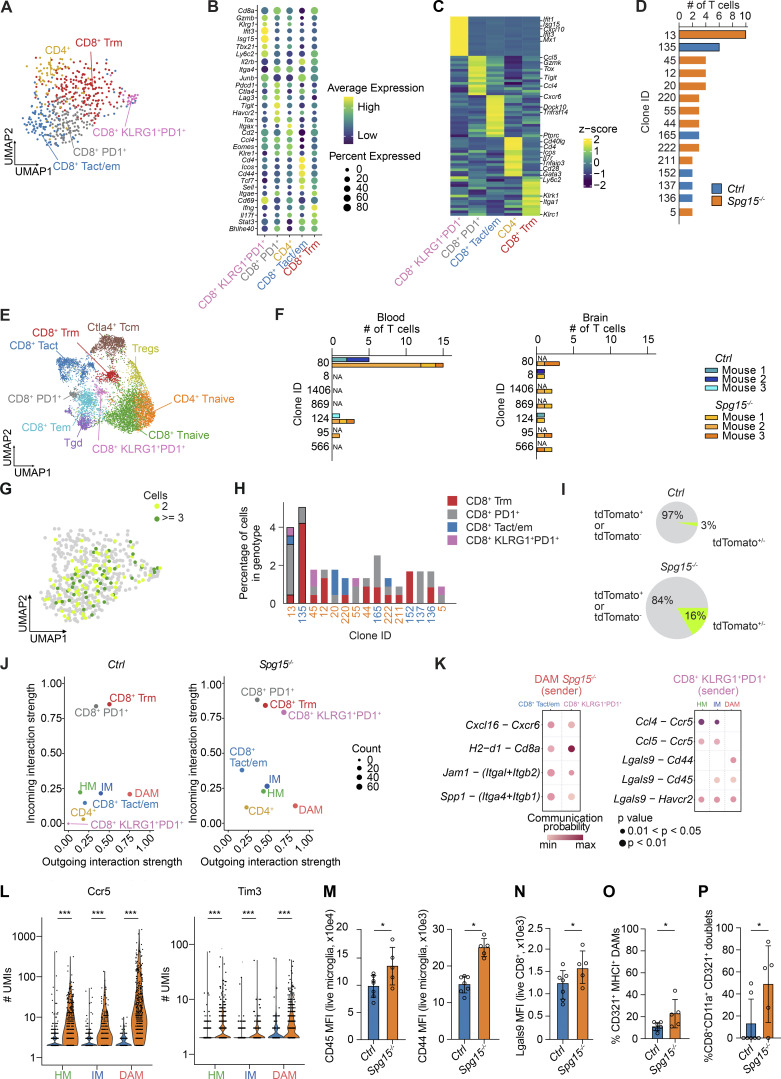
**scRNA-seq analysis reveals expansion of CD8** ^**+**^ **T cells and intricate bidirectional communication between DAM and CD8** ^**+**^ **T cells in the CNS of *Spg15*** ^***−/−***^ **mice. (A)** UMAP from full-length scRNA-seq data generated with Smart-Seq2 from CNS T cells isolated from the CNS of 12–15-mo-old *Ctrl* and *Spg15*^*−/−*^ mice. **(B)** Dotplot visualizing marker genes used for annotation of the clusters in panel A. Genes were selected based on high expression and previous association with CD8^+^ T cell differentiation. **(C)** Pseudobulk heatmap showing upregulated DEGs identified between cell clusters. DEGs were identified using the Wilcoxon rank-sum test. Resulting genes were filtered for a log_2_ fold change >0.1 and genes expressed in at least 30% of cells in the respective clusters. Genes that were used to further characterize the cell clusters were highlighted. **(D)** T cell clones inferred with TRUST4 were quantified with scirpy. Dominant clones in *Ctrl* (135, blue) or *Spg15*^*−/−*^ (13, orange) are highlighted. **(A–D)***n* = 4 mice for *Ctrl* and *n* = 3 mice for *Spg15*^*−/−*^ on one experimental day. **(E)** UMAP of 3′ scRNA-seq data generated with BD Rhapsody from peripheral blood and CNS T cells isolated from 12- to 15-mo-old *Ctrl* and *Spg15*^*−/−*^ mice. **(F)** T cell clones from the BD Rhapsody data for each mouse in the respective genotypes (*Ctrl*: orange hues, *Spg15*^*−/−*^: blue hues) were quantified with scirpy. **(E and F)***n* = 7 mice for *Ctrl* and *n* = 5 mice for *Spg15*^*−/−*^ on one experimental day. **(G)** Expanded clones were mapped onto the UMAP of Smart-Seq2 data the to visualize transcriptional similarity of expanded clones. **(H)** Compositional analysis of clones. Percentage of the clones from the respective genotype (blue: control; orange: *Spg15*^*−/−*^). The cell cluster identity is color-coded. Dominant clones in *Ctrl* (Clone ID 135, blue) and *Spg15*^*−/−*^ animals (Clone ID 13, orange) are highlighted. **(I)** Percentage of either tdTomato^+^ or tdTomato^−^ CD8^+^ T cell clones (grey) as well as CD8^+^ T cell clones containing both tdTomato^+^ and tdTomato^−^ cells (green) in old *Ctrl* or *Spg15*^*−/−*^ animals. The size of the pies represents the relative number of cells in the respective condition. **(G–I)***n* = 4 mice for *Ctrl* and *n* = 3 mice for *Spg15*^*−/−*^ on one experimental day. **(J)** Inference of cell–cell interaction using CellChat contrasting incoming (y-axis) and outgoing interaction strengths (x-axis, arbitrary unit) visualized for microglia and T cells from mouse brains from *Ctrl* (left) and *Spg15*^*−/−*^ animals (right). The number of interactions was coded as the dot size of the cell cluster. Clusters with drastic changes in communication behavior were highlighted with arrows in the *Spg15*^*−/−*^ (right) plot. **(K)** Ligand–receptor pairs between (left) DAM (sender) and CD8^+^ Tem or KLRG1^+^PD1^+^ CD8^+^ T cells (receiver) and (right) KLRG1^+^PD1^+^ CD8^+^ T cells as sender with HM, IM, and DAM subtypes as receivers. Communication probability was color-coded on a continuous scale. **(L)** Ccr5 and Tim3 expression represented as the number of UMIs derived from Total-seq data was visualized for microglia subsets. *n* = 3 mice per genotype on one experimental day. Statistical significance was assessed using a Wilcoxon rank sum test. **(M)** Median fluorescence intensity (MFI) of CD45 and CD44 in all microglia subsets. **(N)** MFI of Lgals9^+^ on total CNS CD8^+^ T cells. **(O)** Percentage of CD321^+^ MHCI^+^ expressing DAM. **(P)** Percentage of CD8^+^ CD11a^+^ T cell CD321^+^ microglia doublets among all doublets which were acquired from spinal cords (SCs) of either *Ctrl* (blue) or *Spg15*^*−/−*^ (orange) animals. **(M–P)***n* = 7 mice for *Ctrl* and *n* = 5 mice for *Spg15*^*−/−*^ on two experimental days. Data are represented as mean with SD, and statistical significance was assessed using the Mann–Whitney *U* test, and *P < 0.05, ***P < 0.001.

To characterize T cell differentiation in *Spg15*^*−/−*^ mice, we performed Smart-Seq2 transcriptomics of CD3^+^ cells (Picelli et al., 2014), yielding 526 high-quality αβ T cells. UMAP analysis identified four distinct CD8^+^ T cell clusters (Fig. 5 A and Fig. S2 Q), corresponding to four of the clusters of tissue-resident effector-like CD8^+^ T cells identified by flow cytometry (Fig. 4 J), while CD4^+^ T cell were too few for further subtyping (Fig. 5 A and Fig. S2 Q). Marker gene analysis, including genes differentiating effector CD8^+^ T cell states (Fig. 5 B), confirmed a KLRG1^+^ PD1^+^ subset (*Pdcd1*, *Tbx21*, *Ly6c2*, *Ifit3*, *Gzmb*, and *Klrg1*) (Hussain and Quinn, 2019; Zöphel et al., 2022; Istaces et al., 2019) (Fig. 4 J), a PD1^+^ effector state of CD8^+^ T cells (CD8^+^PD1^+^) expressing multiple inhibitory receptors (*Pdcd1*, *Ctla4*, *Lag3*, *Tigit*, and *Havcr2*/TIM-3) and *Tox* (E Hirbec et al., 2017; Wherry and Kurachi, 2015), an activated/effector memory CD8^+^ T cell (Tact/em) cluster (*Itgax*, *Eomes*, *Klre1*, and *Cd69*) (Shinoda et al., 2012; Chen et al., 2023), a CD8^+^ tissue-resident (Trm) cluster expressing *Itgae*, *Cd69*, *Rorgt*-associated genes (*Stat3*, *Il17f*), *Ifng,* and *Bhlhe40* (Hussain and Quinn, 2019; Zöphel et al., 2022; Istaces et al., 2019), and a small CD4^+^ T cell cluster (Fig. 5, A and B).

Differential gene expression analysis revealed subset-specific signatures (Fig. 5 C). CD8^+^ Trm highly expressed *Itga1*, *Ly6c2*, *and Klrc1*, indicating long-term memory, tissue residency, repeated stimulation, and cell division (Mackay et al., 2013; Siracusa et al., 2019; Cerwenka et al., 1998; Walunas et al., 1995; Borst et al., 2022). CD8^+^ Tact/em showed expression of canonical activation/effector genes and *Ptprc* and *Tnfrsf14*, while PD1^+^ CD8^+^ T cells expressed activation/effector markers, PD1-associated markers, and *Tox*. The KLRG1^+^PD1^+^ CD8^+^ T cell subset displayed a type-I IFN signature (Fig. 5 C) (Schetters et al., 2018; Chen et al., 2023; Ritzel et al., 2016), suggesting a role in microglia activation. UMAP mapping of tdTomato expression mirrored flow cytometry, with for example, KLRG1^+^PD1^+^CD8^+^ T cells being enriched in tdTomato^+^ cells (Fig. S2, R and S).

In summary, effector-like CD8^+^ T cells expand in the CNS of *Spg15*^*−/−*^ mice, mirroring findings in *Spg11*^−/−^ models (Hörner et al., 2022). Given their early recruitment and local proliferation, it is conceivable that the CD8^+^ T cell pool, both recruited early and late, likely contributes to neuroinflammation and neurodegeneration in *Spg15*^−/−^ mice.

### Expansion of effector CD8^+^ T cells in *Spg15*^*−/−*^ mice is independent of their transcriptional state and time-point of recruitment

**Figure S3.**
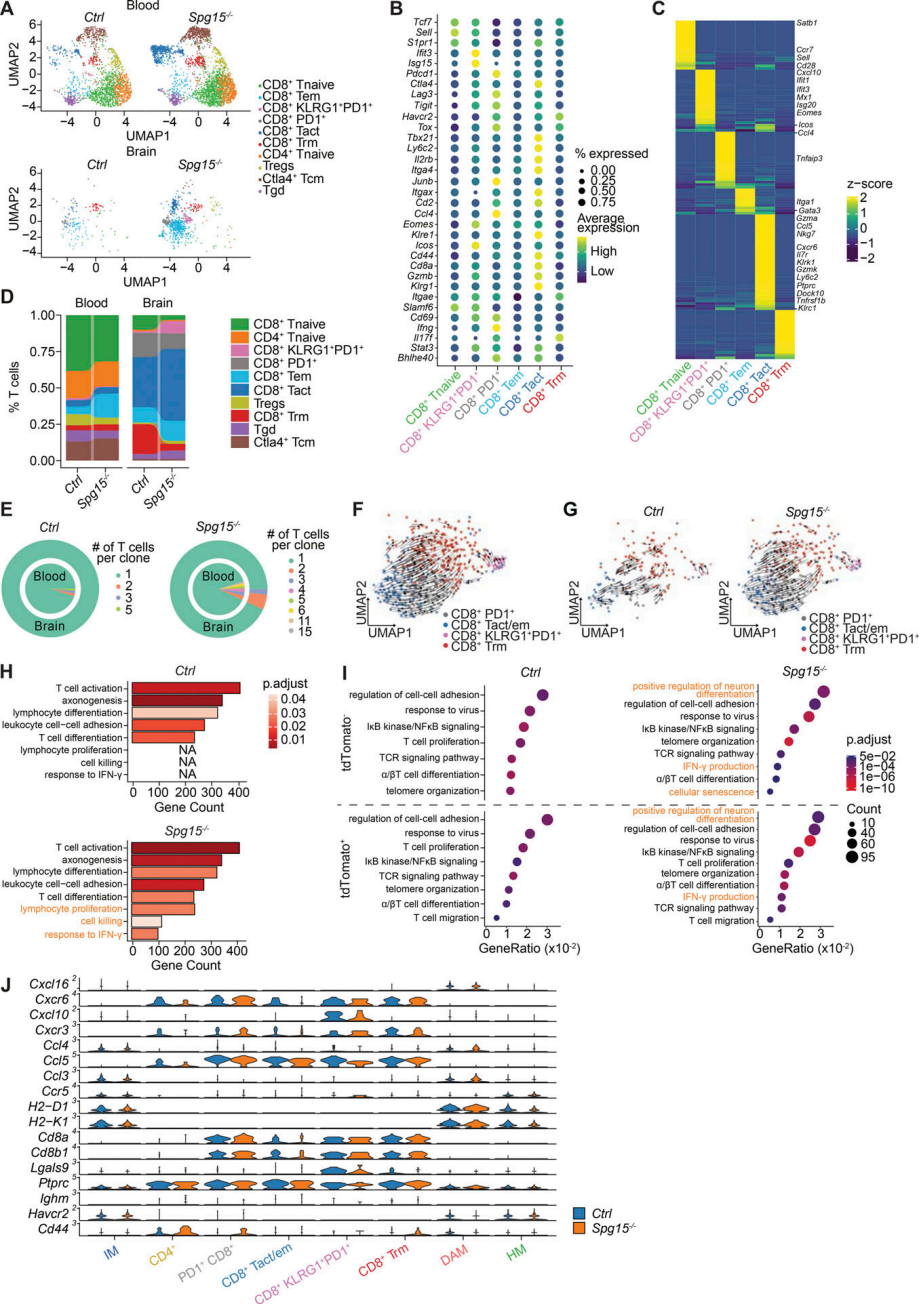
**High-dimensional flow cytometric analysis of the T cell compartment, as well as scRNA-seq, TCR analysis, and tdTomato tracing of the clonal expansion of CD8** ^**+**^ **T cells in the CNS of old *Spg15*** ^***−/−***^ **mice. (A)** UMAP from peripheral blood and CNS T cells isolated from old (12–15 mo) *Ctrl* and *Spg15*^*−/−*^ animals split by organ (left: blood and right: brain) and genotype. **(B)** Dotplot visualizing marker genes used for annotation of CD8^+^ T cell clusters in Fig. 5 E. Genes were selected based on high expression and previous association with CD8^+^ T cell differentiation. **(C)** Pseudobulk heatmap showing upregulated DEGs identified between CD8^+^ T cell clusters. DEGs were identified using the Wilcoxon rank-sum test. Resulting genes were filtered for a log_2_ fold change >0.1 and genes expressed in at least 10% of cells in the respective clusters. Genes that were used to further characterize the cell clusters were highlighted. **(D)** Compositional changes of T cell subtypes as defined in Fig. 5 E in peripheral blood (left) and brain (right) of *Ctrl* and *Spg15*^−/−^ mice. **(A–D)***n* = 6 mice for *Ctrl* and *n* = 5 mice for *Spg15*^*−/−*^ on four experimental days. **(E)** Relative abundance of clonally expanded cells in the peripheral blood or brain of *Ctrl* (left) or *Spg15*^*−/−*^ (right) animals represented as pie charts. Number of cells harboring the same TCR is color-coded. **(F and G)** Averaged velocity vectors, representing the direction and strength of development based on the ratios of exons and introns, were calculated on CD8^+^ T cells only and plotted onto the UMAP from Fig. 5 A (A) or Fig. S2 Q (B). **(H)** DEGs between CD8^+^ T cells from *Ctrl* and *Spg15*^*−/−*^ animals were used to perform GO enrichment analysis and enriched GO terms were plotted. Terms uniquely enriched in *Spg15*^*−/−*^ animals were highlighted in orange. **(I)** Enriched GO terms discovered by GO enrichment analysis using DEGs between either tdTomato^+^ (top) or tdTomato^−^ CD8^+^ T cells (bottom) from *Ctrl* (left) and *Spg15*^*−/−*^ (right) animals. Terms uniquely enriched in *Spg15*^*−/−*^ animals were highlighted in orange. For H and I, a hypergeometric test with all genes in the “biological process” database as background, for statistical enrichment testing, in combination with the Benjamini-Hochberg procedure for multiple testing correction was performed. Ontology terms were filtered for a q-value <0.2 and biological significance. **(J)** Normalized expression of inferred ligand receptor pairs, identified by CellChat, visualized as violin plots for microglia and T cell subsets. Blue color indicates expression in *Ctrl* and orange color in *Spg15*^*−/−*^ animals. DAM: disease-associated microglia; HM: homeostatic microglia; IM; intermediate microglia; Trm: resident memory T cells; Tem: effector memory T cells; Tregs: CD4^+^ regulatory T cells; CD8^+^ activated: activated CD8^+^ T cells; CD8^+^ PD1^+^: PD1^+^ CD8^+^ T cells; CD8^+^ KLRG1^+^PD1^+^: KLRG1^+^PD1^+^ CD8^+^ T cells; Tgd: gamma-delta T cells; CTLA4^+^ Tcm: CTLA4^+^ central memory T cells.

Since CD8^+^ T cells actively contribute to neuroinflammation in SPGs (Hörner et al., 2022), we investigated clonal expansion in *Spg15*^*−/−*^ CNS CD8^+^ T cells. TRUST4 analysis of Smart-Seq2 data (Song et al., 2021) revealed more T cell clones with more than three cells in *Spg15*^−/−^ than *Ctrl* (Fig. 5 D), with one dominant clone in each genotype. To confirm this observation and compare CNS versus peripheral T cells, we performed microwell-based scRNA-seq of CD3^+^ T cells from CNS and blood. UMAP analysis identified 10 T cell clusters (Fig. 5 E), including six CD8^+^ T cell clusters (Fig. 5 E and Fig. S3A) matching prior findings (Fig. 4 J and Fig. 5 A). The T cell compartment in the CNS was dominated by CD8^+^ T cells (Fig. 5 E and Fig. S3 A). We validated T cell clusters and marker genes, including differentially expressed genes for the respective effector CD8^+^ T cell states (Fig. S3, B–D).

Reconstruction of the T cell receptor repertoire (Ulbrich et al., 2023) further confirmed CNS-restricted clonal expansion (Fig. S3 E) with shared clones among *Spg15*^*−/−*^ mice (Fig. 5 F) and shifted V region usage, while usage in the blood and CNS of *Spg15*^*−/−*^ animals was similar (Table S2).

Mapping expanded clones onto the UMAP showed no enrichment in specific CD8^+^ T cell subsets (Fig. 5 G), suggesting TCRs did not dictate transcriptional states. Expanded clones contained diverse differentiation states (Fig. 5 H), with both tdTomato^+^ and tdTomato^−^ CD8^+^ T cells being present (Fig. 5 I). Given the limited number of expanded clones over both datasets, no shared TCRs between datasets were detected, implying the absence of highly abundant CNS antigen-specific TCRs. In summary, *Spg15*^*−/−*^ mice exhibit CNS clonal expansion of effector CD8^+^ T cells, likely driven by alternative recruitment and expansion cues rather than antigen specificity.

### Increased intercellular communication between microglia and T cells in *Spg15*^−/−^ mice

Trajectory analysis revealed two differentiation pathways. Trm cells transitioning over recently activated PD1^+^ T cells toward CD8^+^ Tact/em cells and Trm cells differentiating into KLRG1^+^PD1^+^ CD8^+^ T cells (Fig. S3, F and G). While the Trm to CD8^+^ Tact/em cells transition occurred in both genotypes, *Spg15*^−/−^ CD8^+^ T cells showed enriched GO terms linked to IFN-γ response, proliferation, and cytotoxicity, supporting that *Spg15*^−/−^ CD8^+^ T cells were more activated (Fig. S3 H). Independent of tdTomato expression, *Spg15*^*−/−*^ CD8^+^ T cells exhibited increased IFN-γ-related and neuron differentiation-associated gene expression (Fig. S3 I), suggesting that CD8^+^ T cells exert an active role in intercellular communication in the CNS of *Spg15*^−/−^ mice independent of the time of CNS entry (Fig. S3 I).

To explore CD8^+^ T cell–microglia communication, we used CellChat (Jin et al., 2021) and jointly mapped the microglia and T cell datasets onto a shared two-dimensional manifold and inferred intercellular communication networks (Fig. 5 J). Microglia transitioning from HM via IM to DAM exhibited enhanced signaling toward Trm and PD1^+^ CD8^+^ T cells. Additionally, in *Spg15*^*−/−*^ mice, CD8^+^ Tact/em cells and KLRG1^+^PD1^+^ CD8^+^ T cells acted as strong receivers for signals emanating from DAM in *Spg15*^*−/−*^ mice. This signaling included *Cxcl16*–*Cxcr6*, MHC class I (*H2-d1*)–TCR (*Cd8a*), *Jam1* (CD321)–integrin α1β2 (CD11a), and osteopontin (*Spp1*)–integrin α4β1 interactions (Fig. 5 K and Fig. S3 J). KLRG1^+^PD1^+^ CD8^+^ T cells also influenced HM-to-DAM differentiation via chemokines (*Ccl4*–*Ccr4* and *Ccl5*–*Ccr5*) and galectins (Galectin 9 (*Lgals9*)–Tim3 (*Havcr2*)/*Cd45*/*Cd44*). Total-seq analysis confirmed higher expression of Ccr5 and Tim3 in all microglia subsets in *Spg15*^*−/−*^ mice (Fig. 5 L). Flow cytometry further revealed increased expression of Lgals9-binding receptors CD45 and CD44 in microglia (Fig. 5 M) and elevated Lgals9 levels on CD8^+^ T cells in *Spg15*^−/−^ mice (Fig. 5 N), suggesting enhanced Lgals9-dependent CD8^+^ T cell–microglia interactions. To further assess DAM-CD8^+^ T cell interactions, we quantified CD321^+^ MHC-I^+^ DAMs, which were significantly enriched in *Spg15*^−/−^ mice (Fig. 5 O). Imaging-based flow cytometry confirmed a higher frequency of CD11a^+^ CD8^+^ T cells/CD321^+^ microglia doublets in *Spg15*^−/−^ mice (Fig. 5 P). These findings highlight a microglia–CD8^+^ T cell communication network that likely contributes to neuroinflammation in *Spg15*^*−/−*^ mice.

### Concluding remarks

Immunomodulatory and genetic interventions, including the deletion of CD8^+^ T cells, slow disease in *Spg11*^*−/−*^ mice, highlighting neuroinflammation as a key factor in complicated HSP (Hörner et al., 2022). Here, we provide the first comprehensive immunophenotyping of *Spg15*^*−/−*^ mice. Unlike brain injuries and inflammatory CNS disorders, disease progression in *Spg15*^−/−^ mice is not linked to peripheral myeloid infiltration. Instead, microglia throughout the CNS adopt a DAM-like state, losing homeostatic functions, which is associated with local expansion of early-infiltrating T cells, recruitment of late-generated T cells, as well as prominent activation of CD8^+^ T cells, while other immune cells such as NK cells and B cells remain scarce. Further direct functional characterization of DAM-like microglia and CD8^+^ T cells in *SPG15*^*−/−*^ mice with *ex vivo* approaches will be one pathway to further re-enforce our findings. Moreover, the characteristics and functional importance of activated microglia and CD8^+^ T cells could be validated with human SPG15 postmortem brain tissues or patient-derived iPSC models. Organoid models would also allow us to model how CD8^+^ T cells functionality changes over time when exposed to microglia in the context of SPG15 pathology. Although direct causality is not yet proven, the widespread neuroinflammation we observed is likely driven by the potential interaction of microglia and CD8^+^ T cells and may underlie sensory, mental, and motor impairments. Future studies focusing on restoring microglia homeostatic functions by targeting DAM-like genes, for example, *Apoe*, *C1qb*, depleting CD8^+^ T cells in the brain with genetic and antibody approaches, and blocking the interaction between DAM-like microglia and CD8^+^ T cells will shed light upon the causal roles of microglia and CD8^+^ T cell in Spg15 pathology. Nevertheless, our findings suggest a microglia–CD8^+^ T cell communication network as a potential target for anti-inflammatory therapies, aiming to halt disease progression in complicated HSP.

## Materials and methods

### Animals

Mouse husbandry was in accordance with institutional and EU or national guidelines for animal use, approved by the competent authority (Thüringer Landesamt für Verbraucherschutz, TLV), and supervised by the institutional veterinarians. Animal procedures were performed in adherence to our project licenses issued by the federal state Thüringen (TLV administrative authorization number 02-056/16). Young animals were 2–3 mo old and old animals were between 12 and 15 mo of age. All experimental and breeder mice were on a C57BL/6 background and contained *Cxcr4*^*CreERT2x*^ and *Rosa26*^*tdTomato*^ (Ai14) alleles (Werner et al., 2020). These mice were crossed with *Zfyve26*^−/−^ or *Zfyve26*^+/−^ mice (Khundadze et al., 2013) to obtain *Spg15*^−/−^ (*Zfyve26*^−/−^) and control mice (*Zfyve26*^+/−^; *Zfyve26*^+/+^). All experimental groups contained a similar number of male and female mice. Fluorescent labeling of hematopoietic stem cells was performed by five sequential intraperitoneal injections of 1 mg tamoxifen per day at the age of 4 wk (1 mg/day for five sequential days). Tamoxifen was prepared at 1:10 (wt/vol) in ethanol (5054; Carl Roth) and adjusted to 10 mg ml^−1^ in corn oil (7284; Caelo).

### Beam walk and foot-base angle tests

Motor deficits were assessed using a beam walk test. The beam was 1 m in length and 7 mm broad, with a platform at each end. The beam was mounted 20 cm above the surface of a table. At the far end of the beam, a cage with nesting material from the home cage was placed to attract the animal. Each animal had to cross the beam three times per session. Animals were trained for beam walk starting at the age of 8 mo. After the initial learning phase, the foot-base angle at the toe-off position of the hind paws was measured using single video frames from recordings of mice walking on a beam (Marrone et al., 2022). Gait deficits were assessed once a month. All trials were video-recorded for detailed analysis. The following scoring system was used for assessment: score 0, the animal is sure-footed, hind legs are under the body when walking, tail swings from left to right; score 1, like score 0 but the tail is occasionally nestled on the beam; score 2, the tail is nestled on the beam and shows little movement, hind legs are still under the body, belly is lifted off the beam; score 3, signs like score 4, but not continuously; score 4: tail is nestled on the beam and shows little movement, animal continuously slides with belly on the beam and thus makes a hump, hind legs (one or both) are placed sideways on the beam; and score 5, like score 4 but also forelegs are placed sideways on the beam.

### Immunohistology, microscopy, and image analysis

Mice were killed by 5% isoflurane and then transcardially perfused with PBS followed by 4% formaldehyde/PBS (pH 7.4). Cryosections (40 μm) were cut after cryoprotecting the tissues in 10% and 30% sucrose/PBS. Sections were stored and processed in a 10 mmol/liter Tris, 10 mmol/liter phosphate, and 155 mmol/liter NaCl working buffer (WB), and pH was adjusted to 7.4. Successive 30-min incubations with 50% methanol containing 0.3% H_2_O_2_ and 5% BSA containing 0.3% Triton-X100 were carried out in WB before applying primary antibodies for 24–72 h and fluorescent secondary antibodies for 2 h in WB containing 1% BSA and 0.3% Triton-X100. Images were captured with the LSM 900 using ZEISS ZEN Microscopy Software (RRID:SCR_013672). Images were processed using ImageJ (v1.51, RRID:SCR_003070) and Adobe Photoshop (v21.1.3, RRID:SCR_014199). The following primary antibodies were used: anti-NeuN (Cat# ab177487, RRID:AB_2532109; Abcam), 1:200; anti-CD3 (Cat# ab56313, RRID:AB_940876; Abcam), 1:5,000; anti-P2ry12 (EGT Group Cat# 55043A, RRID:AB_2298886; AnaSpec), 1:500; anti-TCRβ (Cat# 109208, RRID:AB_313431; BioLegend), 1:100; anti-MHC-II (Cat# 107622, RRID:AB_493727; BioLegend), 1:100 or 1:5,000; anti-Iba1 (Cat# ab5076, RRID:AB_2224402; Abcam), 1:400; and anti-Iba1 (Cat# 019-19741, RRID:AB_839504; FUJIFILM Wako Pure Chemical Corporation), 1:1,000. Signal amplification using biotinylated secondary antibodies and fluorescent streptavidin was used to detect MHC-II and CD3. Cell counts and measurement of immunofluorescent signal intensities were performed on confocal images imported into ImageJ. NeuN^+^ cells in the ventral horn of the lumbar spinal cord were counted in a 500 × 500 µm region of interest (ROI) unilaterally placed immediately ventrolateral to the central canal, resulting in NeuN^+^ cells per ventral horn. NeuN^+^ and Iba1^+^ cells in the deep layers of the primary motor cortex were counted in 638 × 638 µm ROIs placed at the corpus callosum/layer VI boundary. Iba1 and P2ry12 signal intensities in the cortex were measured as Integrated Density (IntDen) in a similar ROI and then divided by the area. Iba1 and P2ry12 signal intensities in the ventral horn of the SC were measured as IntDen by selecting the white and grey matter ventrolateral to the central canal as ROI and then divided by the area. CD3 cells were counted in the deep layers of the primary motor cortex and the ventral spinal cord (grey and white matter) in 1 and 0.7 mm^2^ ROIs, respectively. MotiQ (Hansen et al., 2022) was used for morphometric analysis of Iba1^+^ microglia based on a customized Fiji plug-in. MotiQ cropper and a thresholder were used in version v0.1.2 by using Huang for the intensity threshold. MotiQ 3D Analyzer version v0.1.5. was used with a minimum particle volume of 50 voxels. The ramification index is unit-free and was calculated as the ratio between the surface area of the cell and that of a sphere with the same volume and serves as a measure of the complexity of a cellular shape.

### Isolation and analysis of CNS and peripheral blood immune cells

For isolation and analysis of immune cells, the CNS without the olfactory bulb was separated in the cortex, cerebellum, diencephalon, brainstem and lumbar spinal cord, excluding the meninges, and minced in separate tubes. Subsequently, the tissue was cut into small pieces and incubated in a digestion mix (PBS containing 1 mg/ml collagenase D [Roche], 100 U/ml DNase I [Sigma-Aldrich], 2.4 mg/ ml of dispase [Gibco] and 3% fetal calf serum [FCS] [Invitrogen]) for 30 min at 37°C before mechanical disruption through a 100-μm filter on ice. Cells were then enriched using a one-layer 27% Percoll density gradient centrifugation at 600 *g* for 5 min at room temperature with slow deceleration (3/9). Alternatively, for the acquisition with the BD FACSDiscover S8, the brains without olfactory bulb were minced and digested in a digestion mix (PBS containing 1 mg/ml collagenase D [Roche], 100 U/ml DNase I [Sigma-Aldrich], 2.4 mg/ ml of dispase [Gibco] using GentleMACS Octo Dissociator with Heaters [Miltenyi GentleMACS (RRID:SCR_020280]) according to the manufacturer’s recommendation (Tansey, 2024). Cells were then enriched using a two-layer 40%/80% Percoll density gradient centrifugation at 600 *g* for 20 min at 4°C with acceleration and without brake. Peripheral blood was collected via cardiac puncture and incubated with red blood cell lysis buffer (6.5 mM NH_4_C, 0.1 M KHCO_3_, 0.1 mM EDTA, pH 7.4) for 5 min at 4°C to lyse contaminating erythrocytes. For flow cytometry analyses, cells from peripheral blood and the CNS were analyzed with a BD Symphony A5 or a BD FACSDiscover S8, respectively. For florescence-activated cell sorting of cells for scRNA-seq, CD45^+^ brain and peripheral blood cells were directly sorted on a BD Aria III, BD Symphony S6, or BD FACSDiscover S8 cell sorter. The left hemisphere of the brain without an olfactory bulb was used to sort CD45^+^ cells to perform scRNA-seq with SeqWell S^3 (Aicher et al., 2019). The right hemisphere was used for index sorting of CD3^+^ cells into 384-well plates (Penter et al., 2018) for scRNAseq with Smart-Seq2 (Picelli et al., 2014). Only cells isolated with a purity of >98% were used for further analysis. For scRNA-seq and scTCR-seq using the BD Rhapsody system, the whole brain without the olfactory bulb and peripheral blood was used to sort TCRβ^+^ CD11b^−^ T cells.

### Isolation of spinal cord for immunofluorescence staining (IF) and flow cytometry of microglia**–**T cell doublets

For IF stainings, mice were perfused with ice-cold PBS. The spinal cord was gently freed from the spine. To preserve the tissue, the spinal cord was frozen vertically with Tissue-Tek O.C.T (Sakura) on dry ice. To analyze microglia-T cell doublets by spectral flow cytometry (BD FACSDiscover S8), the SC was minced in a digestion mix (DMEM containing DNase I, Collagenase II) and incubated on GentleMACS (Miltenyi Biotec) at 37°C for 20 min, filtered through a 150-μm strainer on ice, and the immune cells were enriched by centrifugation with one layer of 40% isotonic Percoll at 18°C, 900 *g* for 15 min with no acceleration and no brake. The cell pellet was subsequently washed with 30 ml ice-cold PBS, filtered through a 80-μm strainer, and proceeded for antibody staining. Gating based on flow imaging parameters and visual inspection were used to exclude non-interacting doublets and other artifacts.

### Antibodies and flow cytometry analysis

Fluorescent-dye-conjugated antibodies were purchased from Becton Dickinson, BioLegend, or eBioscience (see Table S1). Cell surface staining was performed at 4°C for 20 min with the addition of FcR-blocking reagents (1:100 CD16/32, 2% rat serum). Intracellular staining was conducted using the Foxp3 Staining Buffer Kit (eBioscience) with the addition of FcR-blocking reagents. Data were acquired on a BD LSRII, BD Symphony A5 flow cytometer, or BD FACSDiscover S8 (Becton Dickinson) and analyzed using the FlowJo software package (v10.8.0, RRID:SCR_008520; FlowJo, LLC), cytoflow v1.2, pytometry v0.0.1 (Buttner et al., 2022, *Preprint*), scanpy v1.9.1 (Wolf et al., 2018), scCoda v0.1.4 (Büttner et al., 2021), and cyCONDOR v0.1.3 (Kröger et al., 2024). Briefly, compensation was performed, and cells were gated for CD45^+^ or CD3^+^ (or TCRβ^+^) cells in FlowJo, respectively. The compensated fluorescence values for CD45^+^ or CD3^+^ (or TCRβ^+^) gated cells were exported as .fcs files. These files were imported as Anndata (v0.8.0) objects for high dimensional analysis with Scanpy. After principal component analysis (PCA), UMAP, and Leiden clustering, cell clusters were annotated based on the expression of canonical protein markers. Alternatively, for the data analysis of data on microglia and T cell interaction generated with the BD FACSDiscover S8, CD45^+^ cells were exported as.csv files and imported to cyCONDOR, subsetting each .csv file to 3,000 events. After PCA, UMAP, and FlowSOM (Van Gassen et al., 2015) or Phenograph (Levine et al., 2015) clustering, cell clusters were annotated based on the expression of canonical protein markers. Differential abundance testing was performed using scCODA or GraphPad Prism software eve-8 (RRID:SCR_002798; GraphPad Software). Statistical significance was assessed using the Mann–Whitney *U* test.

### Sciatic nerve sampling and electron microscopy (EM)

After sacrifice, the sciatic nerve of the mice was securely held using forceps at the proximal region (close to the spinal cord) and a clean cut was made on both ends. It was then immediately immersed in EM fixative solution (4% PFA, 2.5% glutharaldehyde, and 20% cacodylate in PBS) and incubated for a month at 4°C. The following steps were performed using a tissue processor EM TP (Leica): rinse 6× for 15 min each in 0.1 M cacodylate buffer. Secondary fixation was performed using 2% osmium + hexacyanoferrate (potassium hexacyanoferrate II) in 0.1 M cacodylate buffer at 4°C for 2 h and then rinsed 4× for 15 min in 0.1 M cacodylate buffer and then 3× for 15 min each with distilled water. Dehydration was performed using an ascending acetone series: 30%, 50%, 70%, 90%, and 95% for 30 min each. Next, samples were immersed in 1% uranyl acetate in 50% acetone for block staining and then washed 3x with 100% acetone for 45 min each. In the meantime, Epon resin was prepared. Samples were transferred to an acetone-resin mixture for 45 min per step in the following ratios: 3:1, 1:1, and 1:3, followed by incubation in pure EPON 3x for 1 h each. Finally, samples were embedded in flat molds for 48 h at 60°C. After thorough polymerization of the samples at the end of the above procedure, they were trimmed using UltraTrim (Leica) followed by acquiring semithin sections of 0.5 µm thickness and placed on glass slides. Methylene blue azure II staining (Richardson et al., 1960) was performed for quality check before proceeding to cut ultrathin sections of 50-nm thickness. Ultrathin sections without poststaining were placed onto copper slot grids coated with a Formvar/Carbon layer for EM analysis. Cutting of both section types was performed using Ultracut S (Leica) and a diamond knife with 35° from DIATOME.

### Imaging and data analysis of electron microscopy—Ultrathin sections

Ultrathin sections were imaged at 5K magnification using JEM 1400 electron microscope (RRID:SCR_020179; JEOL) using an accelerating voltage of 80 kV. The microscope was coupled with an Orius SC 1000 CCD camera (GATAN). Acquired micrographs were analyzed for g-ratio, myelin thickness, and axon diameter in a semiautomated manner using the Myeltracer software as previously described (Kaiser et al., 2021). The software was calibrated to 0.009592 (referring to 1 µm/104.25 pixels) before starting the analysis. Axons and inner and outer myelin sheath borders were outlined manually and the counting was then exported as an Excel sheet. Myelin thickness was calculated by subtracting the inner from outer myelin sheath thickness. The acquired data were later organized into Excel sheets, plotted, and analyzed using GraphPad Prism software (RRID:SCR_000306; GraphPad). For analysis of frequency distributions, data were plotted in percentages to normalize for differences in total counts between different mice.

### Processing of samples for EM after paraffin embedding

The sciatic nerves were transferred into an Excelsior AS tissue processor (Thermo Fisher Scientific) after their fixation in PFA/GA in 40 mM cacodylate buffer as follows. Dehydration: 30 min 50% ethanol at room temperature, 60 min 70% ethanol at room temperature, 60 min 80% ethanol at room temperature, 60 min 95% ethanol at room temperature, and 3 × 60 min 100% ethanol at room temperature. Intermedium: 3 × 60 min 100% xylene at room temperature. Infiltration: 3 × 80 min 100% paraffin at 62°C. Afterward, embedding was done in a paraffin block.

Reprocessing of nerve samples for subsequent EM embedding in resin was performed as follows: paraffin blocks were melted in a 65°C wax bath, and nerves were isolated and carefully transferred into the Leica Tissue Processor EMTP with the following steps. Intermedium: 6 × 20 min 100% xylene at room temperature. Hydration: 3 × 20 min 100% ethanol at room temperature, 1 × 30 min 95% ethanol at room temperature, 2 × 30 min 90% ethanol at room temperature, 2 × 20 min 70% ethanol at room temperature, 2 × 15 min 50% ethanol room temperature, 1 × 15 min 30% ethanol at room temperature, 1 × 15 min H_2_O at room temperature, 1 × 15 min 0.1 M cacodylate buffer at room temperature, and gentle postfixation for 30 min with half concentrated EM fixative. Overnight fixation at 4°C with nerve fixative and normal EM processing in EMTP was performed, as previously described.

### SeqWell S^3 sequencing library preparation for CNS CD45^+^ immune cells

CNS CD45^+^ cells from the brains of C57BL/6 *Spg15*^−/−^*Cxcr4*^*CreERT2*^*; Rosa26*^*tdTomato*^ or *Spg15*^+/−^; *Cxcr4*^*CreERT2*^*; Rosa26*^*tdTomato*^ animals were isolated as described above. These cells were labeled with ADT antibodies (BioLegend) according to the manufacturer’s protocol for TotalSeq-A. 50 μl of cell suspension with 1 × 10^6^ cells were resuspended in staining buffer (2% BSA, Jackson Immuno Research; 0.01% Tween-20; Sigma-Aldrich; 1x DPBS; Gibco), and 5 μl mouse TruStain FcX FcBlocking reagent (Cat# 101319, RRID:AB_1574973; BioLegend) was added. The blocking was performed for 10 min at 4°C. In the next step, 1 μg unique TotalSeq-A antibody was added to each sample and incubated for 30 min at 4°C. After the incubation time, 1.5 ml staining buffer was added and centrifuged for 5 min at 350 *g* and 4°C. Washing was repeated three times. Subsequently, the cells were resuspended in an appropriate volume of 1x DPBS (Gibco), passed through a 40-μm mesh (Flowmi Cell Strainer, Merck) and counted using a Neubauer Hemocytometer (Marienfeld). Cell counts were adjusted, and hashtagged cells were pooled equally. The cell suspension was loaded with 25,000 cells to 1.1 × 10^5^ beads per SeqWell array. Reverse transcription, cDNA amplification, and library generation were performed according to the recommendations from Aicher et al. (2019). The reads were aligned using STAR v2.6.1a_08-27 (Dobin et al., 2013) to the mm10 mouse genome (GRCm38.p5; Release M16 Gencode).

### Smart-Seq2 sequencing library preparation for CNS CD3^+^ T cells

CNS T cells from the brains of C57BL/6 *Spg15*^−/−^; *Cxcr4*^*CreERT2*^; *Rosa26*^*tdTomato*^ or *Spg15*^+/−^; *Cxcr4*^*CreERT2*^; *Rosa26*^*tdTomato*^ animals were isolated as described above. Cells were FACS-sorted into eight 384-well plates containing 2.3 μl lysis buffer (guanidine hydrochloride [50 mM; G3272; Sigma–Aldrich], dNTPs [17.4 mM; N0447; NEB], SMART dT30VN primer [2.2 µM; IDT]), retaining protein expression information for every well to subsequently match with the respective single-cell transcriptomic data in an index sorting approach. Plates were sealed and stored at −80°C until further processing. Smart-Seq2 libraries were finally generated on a Tecan Freedom EVO and Nanodrop II (BioNex) system as previously described (Verstegen et al., 2023). In short, lysed cells were incubated at 95°C for 3 min. 2.7 μl RT mix containing SuperScript II buffer (18064071; Invitrogen), 9.3 mM Dithiothreitol, 370 mM Betaine (B0300; Sigma–Aldrich), 15 mM MgCl_2_ (63069; Sigma–Aldrich), 9.3 U SuperScript II RT (18064071; Invitrogen), 1.85 U recombinant RNase Inhibitor (2313A; Takara), and 1.85 µM template-switching oligo (Eurogentec) was aliquoted to each lysed cell using a Nanodrop II liquid handling system (BioNex) and incubated at 42°C for 90 min and 70°C for 15 min. 7.5 μl preamplification mix containing KAPA HiFi HotStart ReadyMix (7958935001; KAPA) and 2 µM ISPCR primers (IDT) was added to each well, and full-length cDNA was amplified for 16 cycles. cDNA was purified with 1× Agencourt AMPure XP beads (A63882; Beckman Coulter) and eluted in 14 μl nuclease-free water (15667708; Invitrogen). Concentration and cDNA size were checked for select representative wells using a High Sensitivity DNA5000 assay for the Tapestation 4200 (5067-5592; Agilent). cDNA was diluted to an average of 200 pg/μl, and 100 pg cDNA from each cell was tagmented by adding 1 μl TD and 0.5 μl ATM from a Nextera XT DNA Library Preparation Kit (FC-131-1096; Illumina) to 0.5 μl diluted cDNA in each well of a fresh 384-well plate. The tagmentation reaction was performed at 55°C for 8 min before the removal of the Tn5 from the DNA by the addition of 0.5 μl NT buffer per well. 1 μl well-specific indexing primer mix from Nextera XT Index Kit v2 Sets A-D and 1.5 μl NPM was added to each well and the tagmented cDNA was amplified for 14 cycles according to manufacturer’s specifications. PCR products from all wells were pooled and purified with 1× Agencourt AMPure XP beads (Beckman Coulter) according to the manufacturer’s protocol. The fragment size distribution was determined using a High Sensitivity DNA5000 assay for the Tapestation 4200 (Agilent), and library concentration was determined using a Qubit dsDNA HS assay (Thermo Fisher Scientific). Libraries were clustered at 1.4 pM concentration using High Output v2 chemistry and sequenced on a NextSeq500 system SR 75 bp with 2*8 bp index reads. Single-cell data were demultiplexed using bcl2fastq2 v2.20. and pseudo aligned to the mm10 mouse genome (GRCm38.p5; Release M16 Gencode) transcriptome using kallisto v0.44.0 (Bray et al., 2016). The sequences of all oligos used for Smart-Seq2 can be found in the table submitted together with the raw data (GSE244539).

### BD rhapsody sequencing library preparation for peripheral blood and CNS TCRβ^+^ T cells

Prior to cDNA library preparation for the whole transcriptome (WTA) and VDJ libraries, TCRβ^+^ from the peripheral blood and brains of the different mice were pooled, with 12 unique mouse sample tags from the Ms Single Cell Multiplexing Kit (BD Biosciences) labeling the different samples before sorting. Single-cell WTA and VDJ sequencing libraries were prepared using the BD Rhapsody Single-Cell Analysis System (BD Biosciences) according to the manufacturer’s specifications. Libraries were quantified and sequenced on the NovaSeq sequencing platform.

### scRNA-seq analysis of peripheral blood and CNS TCRβ^+^ T cells

The quality of sequencing reads was evaluated using FastQC (RRID:SCR_014583) and MultiQC. Sequencing reads (FASTQ) were mapped to the mm10 genome and sample tags were deconvoluted with the BD Rhapsody WTA Analysis Pipeline v2.2. The Seurat objects generated by the pipeline were imported into R for downstream analysis. From 31,398 genes, only protein-coding genes were kept. Ribosomal or genes that were predicted by genome assembly, but in most cases not associated with a function (gene names starting with “Gm”), were also removed, reducing the number of genes to 18,015. Following the recommendations of Luecken and Theis (2019), 7,808 high-quality cells were identified with 17,155 sufficiently expressed genes.

Sequencing data were normalized, transformed, and scaled using scTransform (Hafemeister and Satija, 2019). After performing PCA, the first 20 principal components (PCs) were used for UMAP and construction of the shared nearest neighbor (SNN) graph. The SNN graph was then used to perform clustering with the Louvain algorithm (Traag et al., 2019). For the clustering resolution, the results from clustree v0.4.3 (Zappia and Oshlack, 2018) and NbClust v3.0 (Charrad et al., 2014) were taken into consideration. For these clusters, the marker genes (one against all) were determined using the Wilcoxon rank sum test (Wilcoxon test) (Haynes, 2013a) with a 0.1 log_2_-fold-change (Log_2_FC) cutoff and a threshold for a minimum of 30% of cells expressing a marker gene in question. Canonical marker genes were used for cluster annotation.

### scRNA-seq analysis of CNS CD45^+^ cells

Unique molecular identifier (UMI) corrected expression matrices were imported into R as Seurat objects using Seurat v4.0.2 (Hafemeister and Satija, 2019). From 407,803 barcodes and 28,441 genes, only protein-coding genes were kept, reducing the number of genes to 17,942. Following the recommendations of Luecken and Theis (2019), 5,378 good-quality cells were identified with 13,123 sufficiently expressed genes. After correcting for ambient genes using the SoupX automatic estimation by Young and Behjati (2020), a further reduction to 12,989 genes was achieved. 2,395 (11,935 genes) of the 5,378 cells were identified as *Spg15*^*−/−*^ and 2,913 (11,512 genes) as *Ctrl*. After removing doublets using DoubletFinder (McGinnis et al., 2019), the total number of cells was reduced to 5,233.

Sequencing data were normalized, transformed, and scaled using scTransform (Hafemeister and Satija, 2019). After performing PCA, the first 21 PCs were used for UMAP and the construction of the SNN graph. The SNN graph was then used to perform clustering with the Louvain algorithm (Traag et al., 2019). For the clustering resolution, the results from clustree v0.4.3 (Zappia and Oshlack, 2018) and NbClust v3.0 (Charrad et al., 2014) were taken into consideration. For these clusters, the marker genes (one against all) were determined using the Wilcoxon rank sum test (Wilcoxon test) (Haynes, 2013a) with a 0.25 log_2_FC cutoff and a threshold for a minimum of 10% of cells expressing a marker gene in question. Canonical marker genes were used for cluster annotation. The protein expression matrix from the spiked-in Total-seq antibodies was added to the processed Seurat object to visualize differential protein expression between genotypes within the defined clusters.

### Gene set enrichment analysis (GSEA)

Gene signatures for microglia were obtained from Hammond et al. (2019). A module score enrichment (Tirosh et al., 2016) was performed on our dataset using these signatures, filtering genes that are present in our expression matrix. 10 expression bins were used. In each bin, 50 control genes were randomly selected as background to perform the signature enrichment against them. All cells with a module score of ≤0 were removed to compare only cells with an enriched DAM signature. Then, the resulting module score was log_2_-transformed and log_2_FC was calculated for each *Spg15*^*−/−*^ cell, comparing each module score to the median module score over all *Ctrl* cells.

For the analysis of the functional phenotype in T cells of *Spg15*^*−/−*^ mice, the genes present in the dataset were ranked by their average expression in the respective genotype. This ranked gene list was then transformed from gene names into EntrezID using clusterProfiler v4.0.5 (Yu et al., 2012; Haynes, 2013b) and the mouse annotation database org.Mm.e.g.,.db v3.12.0 (Carlson, 2020). Then, GSEA was performed using clusterProfiler v4.0.5 (Yu et al., 2012) to determine if GO terms from the GO (Biological Processes) database (Ashburner et al., 2000) were enriched in T cells from *Ctrl* or *Spg15*^*−/−*^ animals. To test the statistical significance of the enriched terms, a hypergeometric test (Yu et al., 2015) in combination with the Bonferroni procedure for multiple testing correction was performed (Yu et al., 2012, 2015; Haynes, 2013b). A cut-off of <0.05 for the adjusted P values was used to select significantly enriched GO terms.

### Gene ontology enrichment analysis (GOEA)

First, all genes present after quality control were transformed from gene names into EntrezID using clusterProfiler v4.0.5 (Yu et al., 2012; Haynes, 2013b) and the mouse annotation database org.Mm.e.g.,.db v3.12.0 (Carlson, 2020). All genes found in the GO database were used as background to test against when performing the hypergeometric test (Yu et al., 2015) for significance testing of the enriched term, in combination with the Bonferroni procedure for multiple testing correction (Yu et al., 2012, 2015; Haynes, 2013b). The differentially expressed genes (DEGs) between genotypes were determined by the Wilcoxon test using a 0.25 log_2_FC cutoff and a threshold for a minimum of 10% of the cells expressing the gene in question. The enrichment was performed for terms of the GO (Biological Processes) using clusterProfiler v4.0.5 (Ashburner et al., 2000; Yu et al., 2012). A cutoff of <0.05 for the adjusted P values was used to select significantly enriched GO terms.

### scRNA-seq analysis of CNS T cells by Smart-Seq2

The transcript abundance files in hdf5 format (Hoefling and Annau, 2020) from the pseudo alignment via kallisto v0.44.0 (Bray et al., 2016) were merged and converted into transcripts per kilobase million (TPM) normalized gene expression matrices, with additional length scaling (“lengthScaledTPM”) and otherwise default parameters via the package tximport v1.18.0 (Soneson et al., 2015). The Ensembl IDs were transformed into gene symbols using biomaRt v2.46.0 (Durinck et al., 2005, 2009). The expression matrix and respective metadata were added to a Seurat object. Additionally, fluorescence intensity values from the index sorting to 384-well plates were exported from the index sort .fcs files as .csv files according to the manufacturer’s recommendation (Penter et al., 2018) were added to the merged Seurat object with dplyr v1.0.7 and rlist v0.4.6.1 (Ren, 2016). Cells in the Seurat object were labeled as tdTomato^+^ or tdTomato^−^ based on the fluorescence intensity of this lineage tracer. The Seurat object was further preprocessed, by keeping only protein-coding genes, and genes for the immune receptors (TCR and B cell receptor). Ribosomal and genes that were predicted by genome assembly, but in most cases not associated with a function (gene names starting with “Gm”), were also removed. The above process resulted in 15,134 genes left from the initial 26,970 genes. Cells with <700 genes were removed from the analysis, resulting in 871 cells from the initial 1,471 cells. 526 cells remained for further analysis after removing CD3-negative cells. Gene expression was normalized to the library size per well, multiplied by 10^4^ and Log1p-transformed, followed by centering and scaling (Hao et al., 2021). The PCA was calculated without truncated singular value decomposition (Hao et al., 2021; Li et al., 2019, *Preprint*). The first 20 PCs were used for SNN and UMAP computation. The clustering was performed using the Louvain algorithm (Traag et al., 2019). The graph from the package clustree v0.4.3 (Zappia and Oshlack, 2018) and the different metrics from the package NbClust v3.0 (Charrad et al., 2014) suggested nine clusters to be the most stable. Clusters were annotated using canonical markers. Differences in the number of UMIs and contamination with *Olfr* genes hindered the annotation of T cell subsets, therefore a regression for these parameters was performed. For the regression of *Olfr* genes, a module score enrichment (Tirosh et al., 2016) was performed. Afterward, 10 clusters were found and annotated using canonical markers. The annotation correlated with protein expression levels from the index sort (Penter et al., 2018). After non-T cell clusters were removed, five T cell subsets were identified using analog and associated genes to the FACS panel for CNS T cells described above.

### TCR analysis

TCR sequences were extracted from fastq files generated with Smart-Seq2 as described above using TRUST4 v1.0.7 (Song et al., 2021). For BD Rhapsody, V(D)J single-cell sequencing data were mapped to the mm10 genome and quantified using the BD Rhapsody WTA Analysis Pipeline v2.2. The resulting adaptive immune receptor repertoire (AIRR) tables (Vander Heiden et al., 2018) were combined with the transcriptome/protein expression matrix from above and analyzed using scirpy v0.11.1. Dominant clones were identified in *Spg15*^*−/−*^ and *Ctrl* and a DEG test was performed. DEGs were used to perform GOEA as described above.

### RNA velocity of CNS T cells

Prior to calculating the velocity, unsorted binary alignment map (Li et al., 2009) files were generated from raw fastq files of each well from the Smart-Seq2 protocol with STAR v2.7.10b (Dobin et al., 2013). Splicing information was extracted from the unsorted binary alignment map files using the docker image asaglam/biotools:21.01 in a Singularity runtime. Snakemake v5.3.1 was applied to sort and index unsorted binary alignment map files using samtools v1.9 (Li et al., 2009) and determine the splicing state of genes using velocyto v0.17.16 (La Manno et al., 2018). The resulting loom files contained two expression matrices for spliced and unspliced gene counts (La Manno et al., 2018; Bergen et al., 2020). These files were converted to anndata objects, concatenated into one object and merged with the anndata object containing the previously processed Seurat object, which was converted to anndata using SeuratDisk v0.0.0.9019. After filtering out CD4^+^ T cells and contaminating cells, 5,000 top-expressed genes and 20 minimally shared counts were selected for the calculation of velocity vectors. The neighborhood graph in scanpy (Wolf et al., 2018) generated using the first 30 PCs and 30 neighbors was taken for calculation for the first- and second-order moments, thus accounting for the probabilistic nature of biological systems (Bergen et al., 2020; La Manno et al., 2018; Fröhlich et al., 2016). A likelihood-based dynamical model, considering individual splicing kinetics, was applied. Initial and terminal states and gene trends were inferred using CellRank v1.5.0 (Lange et al., 2022).

### Trajectory analysis of microglial subtypes

Monocle3 v1.0.0 was used to infer developmental trajectory in the scRNA-seq data of microglial subtypes. Detailed procedures were described by Cao et al. (2019) the first 22 dimensions were used to generate a UMAP on microglia only. A connection matrix was then calculated using a modified partitioned approximate graph abstraction (PAGA) (Wolf et al., 2017, *Preprint*) algorithm followed by significance testing of connections between clusters identified via PAGA and Louvain clustering. A principal graph on the low dimensional space was then learned using a modified SimplePPT algorithm (Mao et al., 2017). Pseudotime was inferred using the node in the homeostatic microglia cluster, which had the highest distance in the UMAP to the intermediate microglia cluster, as the root node.

### Intercellular communication

The analysis of intercellular communication was performed according to the recommendations by Jin et al. (2021) using CellChat v1.1.3 (Jin et al., 2021) for the integrated dataset of microglia and T cell subsets. First, Seurat objects were subsetted for microglia subsets and T cells and integrated using Seurat (Butler et al., 2018). The integrated Seurat object was then split by the genotypes into *Ctrl *and *Spg15*^*−/−*^. All three Seurat objects (*Ctrl*, *Spg15*^*−/−*^, *Ctrl* & *Spg15*^*−/−*^) were converted into Cellchat objects using the normalized and log1p transformed expression matrices (Jin et al., 2021). The expression data was subsetted to keep only known signaling genes. Subsequently, overexpressed ligand and receptor genes were identified by the Wilcoxon rank sum test with a significance level of 0.05. The DEGs were then corrected for noise by calculating the average expression of a gene for each cell group, meaning that the quantiles of the gene expression were summed in a weighted manner. Jin et al. (2021) based the prediction of gene–gene interaction on the protein–protein interaction (PPI) networks from STRINGDb (Szklarczyk et al., 2019), assuming physical interaction between the ligand and receptors, so that law of mass can be applied. For that purpose, they projected the expression profiles of the signaling genes onto the PPI by using the random walk network propagation (Cowen et al., 2017). Based on the weights derived from the networks, an interaction probability (e.g., strength) could be modeled. Statistically significant communication pathways between cell groups were identified by using the permutation test. Furthermore, the social network analysis tool sna (Butts, 2008) was applied to calculate the information flow between cell clusters using the metrics out-degree in-degree flow betweenness and information centrality.

### Experimental design, quantification, and statistical analysis

For the design of the experiments, it was not possible to blind the scientists performing the experiments for the experimental groups because the animals had an obvious phenotype which manifested in the animals’ appearance and behavior. Blinding the scientists performing the analysis was also not possible because of the strong proinflammatory and neurodegenerative phenotype being obvious in all stages of the analysis.

Every reported *n* is the number of biologically independent replicates. No statistical methods were used to predetermine sample sizes; however, our sample sizes are similar to those reported in recently published similar studies (Hörner et al., 2022; Forner et al., 2021). All statistical analyses, except for the analyses of sequencing data, were performed with GraphPad Prism software v5-8 (RRID:SCR_002798; GraphPad Software). When analyzing statistical differences between two groups, Mann–Whitney *U* tests were performed. Statistical differences between three or more groups treated under similar conditions were analyzed by one-way ANOVA with Dunnett’s or Tukey’s multiple comparison tests. Two-way ANOVA were performed when comparing multiple groups in the context of different conditions. Sidak’s or Tukey’s multiple comparison tests were performed depending on the experimental conditions. If the same cells or mice were measured at different time points, repeated measure analysis was performed. P values of <0.05 were considered to be significant (ns indicates not significant P > 0.05). Data are representative of at least two independent experiments with at least three animals per group. Descriptive statistics, the performed statistical tests, as well as the number of samples are stated in the figure legends.

### Online supplemental material

Fig. S1 shows neuronal loss and immunophenotyping of microglia in the CNS of old *Spg15*^*−/−*^ mice. Fig. S2 depicts further analysis of the enriched T cell compartment in the CNS of *Spg15*^*−/−*^ mice. Fig. S3 shows high-dimensional flow cytometry analysis of the T cell compartment, as well as scRNA-seq, TCR analysis, and tdTomato tracing for the clonal expansion of CD8^+^ T cells in the CNS of old *Spg15*^*−/−*^ mice. Table S1 contains genotype, age, and experimental type and dates of the animals used (tab “Animals”), antibody panels used for flow cytometry (tab “SPG15_AllABs”), and Total-seq (“Oligos-Abs”). Table S2 contains additional information about the TCR-seq data generated with SS2 and Rhapsody. Tabs “TCR-QC” contain chain pairing quantification across tissue and genotype. Tabs “Alpha-“ or “Beta-Chain-Usage” contain the quantification of VJ- or VDJ-chain usage for alpha- or beta-chains across genotype and tissue.

## Supplementary Material

Table S1 contains genotype, age, and experimental type and experimental dates of the animals used (tab “Animals”), antibody panels used for flow cytometry (tab “SPG15_AllABs”), and CITE-seq (“Oligos-Abs”).

Table S2 contains additional information about the TCR-seq data generated with SS2 and Rhapsody.

## Data Availability

The complete code for the analysis can be found in the repository https://gitlab.dzne.de/ag-beyer/FrolovHuang_SPG15. The custom R package, which was used for some analysis can be found at https://gitlab.dzne.de/ag-beyer/scrnaseq.analysis. scRNAseq, scTCR-seq, and Total-seq data are available at GEO under GSE244539. Source data are available from the corresponding authors upon reasonable request. Cleaning, dimensionality reduction, clustering, DEG testing, and GOEA were performed on the docker image alefrol94/scrnaseq.analysis:reticulate (https://hub.docker.com/r/alefrol94/scrnaseq.analysis). Any additional packages installed were tracked with the renv package v0.14.0. Quantification and visualization of cell numbers/proportions and TCR analysis were done on the docker image wollmilchsau/Scanpy_scCODA:latest (from November 4, 2021, https://hub.docker.com/r/wollmilchsau/scanpy_sccoda). Velocity analysis was performed using the docker image nvcr.io/nvidia/pytorch:21.08-py3. Trajectory analysis of microglial subsets with Monocle3 (Cao et al., 2019) was performed using the docker image jsschrepping/r_docker:jss_R403_S4cran (https://hub.docker.com/r/jsschrepping/r_docker). Conda environments used for the analysis are saved as yaml files.
